# EHD stability of a cylindrical boundary separating double Reiner–Rivlin fluids

**DOI:** 10.1038/s41598-023-30749-y

**Published:** 2023-03-07

**Authors:** Galal M. Moatimid, Doaa R. Mostapha

**Affiliations:** Department of Mathematics, Faculty of Education, Ain Shams University, Roxy, Cairo, Egypt

**Keywords:** Mathematics and computing, Physics

## Abstract

The major aim of this work is to achieve a mathematical technique to scrutinize the nonlinear instability of a vertical cylindrical boundary separation of two streaming Reiner–Rivlin liquids. The system is portrayed by an unchanged longitudinal electric strength. Furthermore, the action of mass and heat transfer (MHT) and permeable media are also considered. The problem is not only of methodological interest but also of scientific and practical interest. To shorten the mathematical analysis, Hsieh’s modulation together with the viscous potential theory (VPT) is employed. The nonlinear diagram is contingent on tackling the governing linear mechanism along with the nonlinear applicable border restrictions. A non-dimensional process produces several non-dimensional physical numbers. A linear dispersion equation is attained and the stability standards are theoretically governed and numerically established. The nonlinear stability procedure reveals a Ginzburg–Landau formula. Consequently, nonlinear stability stipulations are accomplished. Furthermore, by way of the Homotopy perturbation approach, along with the expanded frequency concept, an accurate perturbed technique of surface deflection is attained theoretically and numerically. To validate the theoretical outcomes, the analytical expression is confirmed through the Rung–Kutta of the fourth order. The stable and unstable zones are signified graphically displaying the influences of several non-dimensional numbers.

## Introduction

Fluid Mechanics involves a field called Electrohydrodynamics (EHD) that studies the effects of electrical strength^1^. It might be regarded as a problem involving fluid motion in an electric field. Since many interesting difficulties in EHD involve the action of liquid movement as well as the action of electric strength, it is fundamentally a mixture of these two fields. The complicated connection of interior, viscous, and electric power is incorporated into EHD. It generates eye-catching phenomena in processing machinery for applications such as drop-sparing and inkjet printing. Rayleigh examined charged droplet stability more than a century ago. Thus, EHD may be observed as a hot topic in Fluid Mechanics. To provide a comprehensive picture of EHD, a review article was created^2^. It looked at the stability between two layers at the interface of dielectric liquids in the presence of voltage and imposed fluid fields. EHD stability of the surface separating two overlapping liquids in confined as well as boundless channels has been the subject of numerous ground-breaking studies^3^. The EHD instability of two accumulated liquids in a channel was investigated by tackling the surface-connected prototype^4^. A uniform longitudinal electric strength was thought to examine the EHD stability of an incompressible viscous liquid in a cylinder using a weakly nonlinear technique^5^. Along with the linear fundamental equations of movement, nonlinear boundary stipulations were used. In-depth research was done on the stability of an interface separating various fluids with relaxation time. Investigations were done on the nonlinear EHD of capillary surface waves^6^. A steady axial electrostatic field was tested to check if it had penetrated the system^7^. Many non-dimensional quantities were created as a result of their method. Scientists now have a solid basis for recognizing how viscous fluids flow in cylinders and become unstable when exposed to electric fields thanks to their research. The stability of two horizontal liquids that were superposed one on top of the other, with a perfect gas in the upper layer and a viscous liquid in the bottom was investigated^8^. Centrifugal and Coriolis forces were taken into account. The system was also impacted by a regular, normal electric field. A vertical cylindrical interface in an EHD instability was addressed^9^. The system under consideration was affected by an even axial electric field. Both the VPF and the conventional normal modes analysis were used. The current investigation is carried out using this methodology in light of how significant the presence of the electric field is.

Heat transference in engines occurs during consumption. This transfer also results in mass transfer as a simultaneous consequence of the fluid petroleum evaporating. Consequently, the presence of MHT must be considered to show the stability of a fluid machine. In many circumstances, including heat transfer in engineering, technology as well as geophysical issues, the MHT across the interface is crucial^10^. Hsieh^11,12^ was the first to provide a simplification of the instabilities of both the Rayleigh–Taylor instabilities (RTI) besides Kelvin–Helmholtz instabilities (KHI) under the influence of (MHT). When there was MHT across the interface, the action of a pivotal magnetic field on the instability of a cylindrical interface was investigated^13^. The MHT was realized to have a stabilizing effect. The instability of the KHI at the surface of two viscous liquids under the action of a tangential magnetic field was investigated^14^. The MHT was revealed to have a destabilizing effect. When there was MHT across the interface, the action of the magnetic field and porous surrounding on the KHI of the jet was investigated^15^. The magnetic field permits a stabilizing impact on the stability configuration. It was revealed that the MHT played a dual role in the stability profile. The MHT was used to analyze nonlinear instability across a cylinder-shaped magnetic fluid contact^16^. It was demonstrated that the nonlinearity expanded the MHT zone of stability. The nonlinear stability method, however, revealed that it played a dual role in the stability configuration. This in-depth investigation was done because the viscoelastic Oldroyd-B model plays an important role in geothermal, engineering, and industrial applications^17^. A method of examining the nonlinear stability of a vertical jet between two Oldroyd-B models was provided. The porous medium and the impact of MHT were supposed, and the technique was affected by an unaltered axial electric field. As is common, there is a direct correlation between mass and the amount of heat transfer. Without heat transfer, this beautiful world of people, animals, jungles, rivers, and seas would not exist, even if the idea of heat replacement from one place to another involves heat transference. The mathematical analysis was condensed using Hsieh's modulation and VPT. Solar energy is transferred to the earth via heat transfer. Therefore, the present task is to tackle both VPT and MHT.

In recent years, both theoretical and experimental studies have given an increasing amount of attention to non-Newtonian fluid flow issues. The relationship between the stress tensor and the various kinematic and thermodynamic variables, often known as the constitutive equation, provides the mathematical description of such a fluid. Non-Newtonian fluids are those that do not conform to Newtonian constitutive relations. Examples include large molecular polymers, which are frequently used in lubricants, nylon, blood, clay, detergents, and paints. Due to the intermolecular trussing strong binding, the tension inside the viscoelastic fluids continue to exist to some extent even after the stress forces are removed. The memory effect was the name given to this unique quality. A brand-new non-Newtonian fluid was developed by Reiner^18^ and Rivlin^19^ and it successfully predicted the flow conduct of various biological besides geological substances, as well as polymers and other food items. Later, the Reiner–Rivlin revealed that rheological connection was insufficient for articulating the effects of common stresses^20^. Numerical analysis was used to study the Reiner–Rivlin fluid within rectangular conduits^21^. This study as an interesting conclusion showed that the presence of the second normal stress significantly boosts heat transference. The impact of suction/injection on Reiner–Rivlin liquid via a spinning permeable disk has been numerically solved^22^. The crucial finding of this study is that, compared to injection, the action of heat transference in the presence of suction is more significant. Another work^23^ investigated the influx of the Reiner–Rivlin liquid within a rotating disk with actions from ion slip and hall current. An intriguing finding of this experiment shows that, compared to any Newtonian liquid, the action of the Ion slip on the pivotal speed is more stable for Reiner–Rivlin liquid. It discussed how to numerically solve the Reiner–Rivlin fluid influx with partial slip caused by a revolving disk^24^. According to this study, larger torque and slip factor values are required to maintain the disk constant rotation. The work was expanded^25^ by talking about the numerical solution of the fluid influx caused by a revolving disk with many slips. One of the main findings of this work is that the Reiner–Rivlin liquid parameter has increased while the radially outward flow has decreased. In recent work, the heat relocation besides entropy generation of a spinning Reiner–Rivlin liquid disk causing Von Karman flow was investigated^26^. The key finding of this research is that the moment coefficient dramatically decreases for the Reiner–Rivlin liquid. Compared to Newtonian fluids, non-Newtonian fluids have fascinating possibilities for heat transmission^27^. Reiner–Rivlin nanofluid influx via a rough spinning disk within heat flux is a novel non-Newtonian liquid that was investigated in a permeable medium. The addition of gyrotactic microorganisms to the nanofluid increases the stability of the nanoparticles.

The foundation of thermal convection for both pure liquids and liquids filling permeable resources has been extensively considered by numerous scientists. This topic has gained remarkable interest for its applications in various fields, including engineering and manufacturing processes (water systems, nuclear waste disposal, heat exchanger technology, and the collection of temperature substances like grain and coal). Numerous different disciplines that include theoretical physics like pore water convection within carbonaceous categories or classes of maternal physiques were considered. The fluid flow across porous media was studied numerically^28^. The beginning of convection in anisotropic permeable media was analyzed^29^. The inception of thermic convection via a horizontal layer of anisotropic, consistently spinning around a perpendicular axis and homogeneously heated from below, was evaluated^30^. Their study was concerned with Darcy’s law in both micropores as well as macropores. They numerically investigated the impact of the revolution and the effect of anisotropy at the beginning of convection. Two models for RTI in porous ambiance (linearized approximation with small disturbances as well as nonlinear approach) were analyzed^31^. The configuration was unstable, and a slight interface disturbance accumulated with time. It was regarded as a flat structure in a porous material subject to Darcy's law. The nonlinear instability analysis of cylindrical Walters' B liquids, where the fluids were entirely filled in permeable media, was carried out^32^. The incentive to examine this field is attributed to the huge consideration it attains in various reasonable circumstances in physics and manufacturing practical. Remember that the present work examines the action of a constant tangential electrostatic field beside the interfacial nonlinear stability with permeability. In view of the relevance of the porous interaction, the current inquiry is carried out utilizing this technique.

Rheological simulations, particularly Reiner–Rivlin Fluids, are receiving increased interest as can be observed from the above-mentioned topics and their significance in many fields. The current article scrutinizes this aspect as well as the nonlinear analysis of stability via cylindrical boundary between two incompressible dielectric liquids through the following approach. There is a regular axial electric field acting on the mechanism. The VPF is taken to condense the mathematical structure. The appropriate nonlinear border requirements frequently affect how the linear equations of movement behave. Consequently, a nonlinear differential equation subsequently results. To epitomize the texture of the article, the remainder of the text is structured as follows: The organization of the theoretical structure is described in "Theoretical methodology", where fundamental equations of motion besides the adequate nonlinear border stipulations are generated. The surface displacement nonlinear equation is also derived. The method of solution is covered in "Method of solution". The investigation of the linear stability technique is shown in “Standard stability analysis”. Section "Nonlinear stability investigation" discusses the nonlinear stability approach. The displacement profile of the interface is covered in "The Surface displacement profile". Additionally, this section contains the consistency between the approximate solution and the numerical one. Finally, "Conclusions" provides a summary of the closing statements based on the key characteristics.

## Theoretical methodology

**Figure 1:**
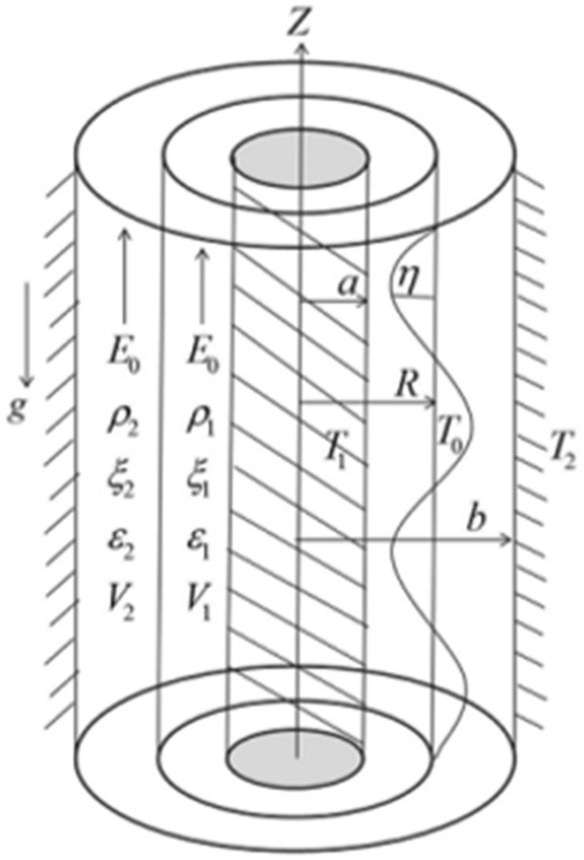
Description of natural prototype of the structure.

Two immiscible fluids are assumed to exist within two solid concentrically vertical cylinders of radii $a,\,b\,(a < b)$ and separated by an undisturbed interface $r = R$. Two perpendicular movements of corresponding incompressible electrified fluids fill the concentric cylinders. The regular and homogeneous liquids are of an unlimited length. The influx streams throughout the porous medium in the presence of porosities and Darcy's factors. These factors rely on the proportion of the liquid viscosity to the movement permeability. The parameters illustrate the portions of the permeable media addressed at the beginning of the paper. Through porous media, Darcy's law governs the influx. The permeability of the two liquids is the same for appropriateness. Darcy's law, an empirical assumption that characterizes the creeping influx of Newtonian fluids through porous media, is corroborated through scientific evidence. The liquids are represented by the Reiner–Rivlin liquid prototype. In this presentation, the subscripts $1$ and $2$ refer to the constraints that are related to the liquid within and out of the stationary boundary, correspondingly. In the undisturbed state, the interior and exterior fluids have constant densities and dielectrics. The two liquids are presumed to be impacted by an unchanged longitudinal electric strength which is implemented adjacent to the positive *z*-path. In light of Hsieh’s simplified construction^11,12^, the rigid boundaries are kept at different regular heats. Additionally, the interface is maintained at another uniform temperature. The acceleration of gravity $g$ acting along the negative *z*-path is considered. The fluids are mainly flowing at regular speeds. For further accessibility, the cylindrical coordinates are assumed. Typically, in the basic situation, the *z*-axis signifies the symmetry structure. The theoretical physical prototype is drawn in Fig. 1.

As shown by Moatimid et al.^33^, the equations of movement through the permeable milieu can be written as:

1 $$\frac{\rho }{m}\left( {\frac{{\partial \underline{v} }}{\partial t} + \frac{1}{m}(\underline{v} \,.\,\nabla )\underline{v} } \right). = - \nabla P - \rho g\underline{e}_{z} - \frac{\xi }{m}\underline{v} .$$

The liquid density is supposed to be consistent. Consequently, the incompressibility restriction becomes

2 $$\nabla .\,\underline{v} = 0.$$

As demonstrated by Reiner^18^ as well as Rivlin^19^, the tensor of Cauchy stress of the Reiner–Rivlin liquid prototype may be written as:

3 $$\underline{{\underline{\tau } }}^{(vis)} = - P\,I + \underline{{\underline{\varsigma } }} ,$$

where

4 $$\underline{\underline{\zeta }} = \gamma \,\underline{\underline{A}} + \beta \,\underline{\underline{A}}^{2}$$

Additionally, the rate of strain tensor is specified as:

5 $$\underline{{\underline{A} }} = \nabla \underline{V} + \nabla \underline{V}^{T}$$

The liquid jet is stable for all asymmetric modes $m \ne 0$, but it is unstable for axisymmetric modes $m = 0$, as proved by numerous earlier research, such as Chandrasekhar^34^. Subsequently, the axisymmetric mode of disturbance is the most intriguing mode of disturbance. Now forward, only the instance $m = 0$ of was taken into consideration moving forward. *Therefore, only radial symmetry was employed.* On the foundation of the previous interpretation, elements of stress tensor are formulated as^33^:

6 $$\left. {\begin{array}{*{20}c} {\tau_{rr}^{(vis)} = - P + 2\gamma \frac{\partial u}{{\partial r}} + \beta \left[ {4\left( {\frac{\partial u}{{\partial r}}} \right)^{2} + \left( {\frac{\partial w}{{\partial r}} + \frac{\partial u}{{\partial z}}} \right)^{2} } \right]} \\ {\tau_{rz}^{(vis)} = \gamma \left( {\frac{\partial w}{{\partial r}} + \frac{\partial u}{{\partial z}}} \right) + 2\beta \left( {\frac{\partial w}{{\partial r}} + \frac{\partial u}{{\partial z}}} \right)\left( {\frac{\partial u}{{\partial r}} + \frac{\partial w}{{\partial z}}} \right)} \\ {\tau_{zz}^{(vis)} = - P + 2\gamma \frac{\partial w}{{\partial z}} + \beta \left[ {4\left( {\frac{\partial w}{{\partial z}}} \right)^{2} + \left( {\frac{\partial w}{{\partial r}} + \frac{\partial u}{{\partial z}}} \right)^{2} } \right]} \\ \end{array} } \right\}.$$

At this point, the rheological performance of the viscoelastic liquid that is described by the Reiner–Rivlin simulation was investigated.

To analyze the stability profile, the cylindrical boundary next to a little disturbance around the basic structure leads to a boundary displacement as:

7 $$r = R + \eta (z;t).$$

Consequently, after a slight but limited perturbation of the basic situation, the boundary shape might be represented as:

8 $$S(r,z;t) = r - R - \eta (z,t).$$

The unit boundary outwards the perpendicular vector can be expressed as:

9 $$\underline{n} = \nabla S/\left| {\nabla S} \right| = (\underline{e}_{r} - \,\eta_{z} \,\underline{e}_{z} )(1 + \eta_{z}^{2} )^{ - 1/2} \,\,\,,$$

Zero-order formula of Eq. (1) produces,

10 $$P_{0j} = - \rho_{j} gz - \frac{{\xi_{j} }}{{m_{j} }}V_{j} z + \Gamma_{j} (t),$$

The disturbed velocity is designated as follows:

11 $$\underline{v}_{j} = V_{j} \underline{e}_{z} + \nabla \varphi_{j} = \frac{{\partial \varphi_{j} }}{\partial r}\underline{e}_{r} + \left( {V_{j} + \frac{{\partial \varphi_{j} }}{\partial z}} \right)\underline{e}_{z} .$$

Owing to the incompressibility stipulation, the potential function $\varphi_{j} (r,z;t)$ must satisfy the following Laplace formula:

12 $$\nabla^{2} \varphi_{j} = 0\,.\,$$

The explanations of supply functions will be expressed by the normal mode evaluation. Then**,** the potential $\varphi_{j} (r,z;\,\,t)$ might be formulated as:

13 $$\varphi_{j} (r,z;t) = \hat{\varphi }_{j} (r;t)e^{ikz} + c.c.\,,$$

The finite solutions of Laplace’s equation can be designated as

14 $$\hat{\varphi }_{1} (r,t) = C_{1} (t)\,I_{0} (kr) + D_{1} (t)\,K_{0} (kr)\,,$$

and

15 $$\hat{\varphi }_{2} (r,t) = C_{2} (t)\,I_{0} (kr) + D_{2} (t)\,K_{0} (kr)\,.$$

The time-related functions may be governed by the applicable nonlinear border restrictions.

The integration of the linear equation of movement (1) provides Bernoulli’s formulation, which guarantees the function of the pressure as^33^:

16 $$P_{j} = - \frac{{\rho_{j} }}{{m_{j} }}\left( {\frac{{\partial \varphi_{j} }}{\partial t} + \frac{{V_{j} }}{{m_{j} }}\varphi_{j} } \right) - \frac{{\xi_{j} }}{{m_{j} }}\varphi_{j}$$

In addition, as exhibited by Melcher and Taylor^1^, the quasi-static estimation is utilized in the Maxwell equations by disregarding the effect of the magnetic field. Consequently, these equations may be written as

17 $$\nabla .(\varepsilon_{j} \underline{E}_{j} ) = 0.$$

and

18 $$\,\nabla \times \underline{E}_{j} = \underline{0}.$$

As provided in the structure of the model, no surface currents are presumed. Henceforth, the electric strength might be formulated in the expression of scalar electro-static potentials $\,\psi_{j} (r,z;t)$. i.e., $\underline{E}_{j} = E_{0} - \nabla \psi_{j} (r,\,z;t)$**.** So, the electric strengths may be expressed as:

19 $$\,\underline{E}_{j} = - \frac{{\partial \psi_{j} }}{\partial r}\underline{e}_{r} + \left( {E_{0} - \frac{{\partial \psi_{j} }}{\partial z}} \right)\,\underline{e}_{z} .$$

The electrical insulating liquid validates the static structure of Maxwell equations that provide Laplace’s equation of the potential $\,\psi_{j} (r,z;t)$. Therefore, the mixture of Eqs. (17–19) generates,

20 $$\nabla^{2} \psi_{j} = 0.$$

One can write the electrical potentials as:

21 $$\hat{\psi }_{1} (r,t) = A_{1} (t)\,I_{0} (kr) + B_{1} (t)\,K_{0} (kr)\,,$$

and

22 $$\hat{\psi }_{2} (r,t) = A_{2} (t)\,I_{0} (kr) + B_{2} (t)\,K_{0} (kr)\,.$$

The time-supported functions will be calculated by tackling the applicable nonlinear border stipulations.

### Boundary restrictions of nonlinear approach

Using the suitable nonlinear boundary restrictions and disregarding the nonlinear portions of the controlling equations of movement are the primary components of the weak nonlinear description, as was already indicated. At this point, the nonlinear boundary requirements should be achievable in order to present the boundary-value problem. It is essential to display the problem typical stress tensor. The Reiner–Rivlin type stress tensor and the EHD stress make up the elements of this stress tensor. The interface boundary restrictions, however, are the focus of the second stress. The following two categories will be used to separate these conditions:

#### At the solid boundaries

The fluid normal speeds should cease at both stiff barriers, resulting in the following instances^33^:

23 $$\frac{{\partial \varphi_{2} }}{\partial r} = 0\,,\,\,\,\,\,\,\,\,\,{\text{at}}\,\,\,\,\,\,\,r = b,$$

and

24 $$\frac{{\partial \varphi_{1} }}{\partial r} = 0\,,\,\,\,\,\,\,\,\,\,{\text{at}}\,\,\,\,\,\,\,r = a,$$

As usual in the EHD stability problems. In the case of a perpendicular electric, the tangential component of the perturbed electric field should be disappeared at the rigid boundaries, and vice-versa for the tangential electric field. For this purpose, two recent references have been added to incorporate the normal as well as the tangential fields^8,9^. Consequently, the normal components of the electric potential also need to disappear at the boundaries, which requires the following cases:

25 $$\frac{{\partial \psi_{2} }}{\partial r} = 0\,,\,\,\,\,\,\,\,\,\,{\text{at}}\,\,\,\,\,\,\,r = b,$$

and

26 $$\frac{{\partial \psi_{1} }}{\partial r} = 0\,,\,\,\,\,\,\,\,\,\,{\text{at}}\,\,\,\,\,\,\,r = a,$$

#### At the free boundary $\,r = R + \eta (z;t)$

(i) By utilizing the shortened modulation of Hsieh^11,12^, the preservation of mass through the boundary between the two liquids requires:

27 $$\,\left\| {\rho_{j} \left( {\frac{\partial S}{{\partial t}} + \frac{1}{{m_{j} }}(\underline{v}_{j} \,.\,\nabla )S} \right)} \right\| = 0\,\,\,\,\,{\text{at}}\,\,\,\,\,\,\,r = R + \eta .$$

where $\left\| * \right\| = *_{2} - *_{1}$ is a function around the boundary.

(ii) Hsieh’s^11,12^ postulated that the quantity of the distributed hidden temperature relates to the immediate location of the boundary. Consequently, the interface stipulation for energy is formulated as follows:

28 $$\,L\rho_{1} \left( {\frac{\partial S}{{\partial t}} + \frac{1}{{m_{1} }}(\underline{v}_{1} \,.\,\nabla )S} \right) = 0\,\,\,\,\,{\text{at}}\,\,\,\,\,\,\,r = R + \eta .$$

In order to consider the steady state, the two significant radial heat fluxes in the two regions 1 and 2 are $K_{1} (T_{1} - T_{0} )/R\ln (R/a)$ and $K_{2} (T_{0} - T_{2} )/R\ln (b/R)$, respectively.

As indicated by Hsieh^11,12^, it follows that

29 $$F(\eta ) = \frac{{K_{2} (T_{0} - T_{2} )}}{(R + \eta )(\ln b - \ln (R + \eta ))} - \frac{{K_{1} (T_{1} - T_{0} )}}{(R + \eta )(\ln (R + \eta ) - \ln \,b)} .$$

By employing Maclaurin series, Eq. (29) is extended near $\eta = 0$ as:

30 $$F(\eta ) = F(0) + \eta \,F^{\prime}(0) + \frac{1}{2!}\eta^{2} F^{\prime\prime}(0) + \frac{1}{3!}\eta^{3} F^{\prime\prime\prime}(0) + \cdots .$$

For the basic situation $F(0) = 0$, the following relationship is considered:

31 $$\frac{{K_{2} (T_{0} - T_{2} )}}{(\ln b - \ln R)} = \frac{{K_{1} (T_{1} - T_{0} )}}{R(\ln R - \ln \,a)} = G ,$$

where G signifies the heat flux through the boundary connecting the two liquids.

Substituting from Eqs. (8), (11) and (29)–(31) into Eq. (28), one finds

32 $$\rho_{1} \left[ { - \frac{\partial \eta }{{\partial t}} + \frac{1}{{m_{1} }}\left( {\frac{{\partial \varphi_{1} }}{\partial r} - V_{1} \frac{\partial \eta }{{\partial z}} - \frac{{\partial \varphi_{1} }}{\partial z}\frac{\partial \eta }{{\partial z}}} \right)} \right] = \alpha_{1} \left( {\eta + \alpha_{2} \eta^{2} + \alpha_{3} \eta^{3} } \right)\,\,,\,\,\,{\text{at}}\,\,\,r = R + \eta,$$

where the constants $\alpha_{1} ,\,\alpha_{2} \,,\,{\text{and}}\,\,\alpha_{{3}}$ may be listed as follows:

$$\alpha_{1} = \frac{G}{R\,L}\left( {\frac{1}{{\ln \left( {{b \mathord{\left/ {\vphantom {b R}} \right. \kern-0pt} R}} \right)}} - \frac{1}{{\ln \left( {{a \mathord{\left/ {\vphantom {a R}} \right. \kern-0pt} R}} \right)}}} \right),\,\,\,\,\alpha_{2} = \frac{1}{2\,R}\left( {2\,\left( {\frac{1}{{\ln \left( {{b \mathord{\left/ {\vphantom {b R}} \right. \kern-0pt} R}} \right)}} - \frac{1}{{\ln \left( {{R \mathord{\left/ {\vphantom {R a}} \right. \kern-0pt} a}} \right)}}} \right) - 3} \right)$$

and

$$\alpha_{3} = \frac{1}{{6R^{2} }}\left( {11 - 12\left( {\frac{1}{\ln (b/R)} - \frac{1}{\ln (R/a)}} \right) + 6\left( {\frac{1}{{(\ln (b/R))^{2} }} + \frac{1}{{(\ln (R/a))^{2} }} - \frac{1}{\ln (R/a)\ln (b/R)}} \right)} \right).$$

(iii) The perpendicular element of the electric field at the boundary is continuous.

33 $$\underline{n} \,.\,\left\| {\varepsilon \,\underline{E} } \right\|\, = 0,$$

(iv) In accordance with the non-existence of surface charges, the jump of tangential components of the electric deflection is continuous at the boundary. Therefore, one gets

34 $$\underline{n} \,\, \wedge \,\left\| {\,\underline{E} } \right\|\, = \underline{0} .$$

To examine the stability profile of the structure, the residual boundary requirement necessitates that the perpendicular component of the stress tensor be discontinuous by the surface tension. As stated above, the complete stress tensor is composed of two pieces. The first part is the viscoelastic tensor of Reiner–Rivlin prototype which is provided in Eq. (3). The additional one concerns the stresses of the electric strength that can be formulated as follows:

35 $$\tau_{ij}^{(elec)} = \varepsilon E_{i} E_{j} - \frac{1}{2}\varepsilon E^{2} \delta_{ij}.$$

Therefore, the total stress tensor may be expressed as

36 $$\tau_{ij} = \tau_{ij}^{(elec)} + \tau_{ij}^{(vis)} .$$

(v) Lastly, the mechanisms of the stresses contain hydrodynamic pressure, surface tension, MHT effects, viscoelastic stresses, and electric stresses. Therefore, one finds

37 $$\begin{gathered} \,\left\| {\frac{\rho }{m}\left( {\underline{v} \,.\,\nabla S} \right)\left( {\frac{\partial S}{{\partial t}} + \frac{1}{m}(\underline{v} \,.\,\nabla )S} \right)} \right\| + \left( {\sigma \,\nabla \,.\,\underline{n} } \right)\left| {\nabla S} \right|^{2} - \left( {\frac{\partial \eta }{{\partial z}}} \right)^{2} \left\| {\tau_{zz} } \right\| + 2\frac{\partial \eta }{{\partial z}}\left\| {\tau_{rz} } \right\|\,\,\, - \left\| {\tau_{rr} } \right\|\, + \hfill \\ \left( {1 + \left( {\frac{\partial \eta }{{\partial z}}} \right)^{2} } \right)\,\left\| {\varepsilon \left( {\frac{1}{2}E_{0}^{2} - E_{0} \frac{\partial \psi }{{\partial z}} + \frac{1}{2}\left( {\left( {\frac{\partial \psi }{{\partial z}}} \right)^{2} - \left( {\frac{\partial \psi }{{\partial r}}} \right)^{2} } \right)} \right)} \right\|\, + 2\,\frac{\partial \eta }{{\partial z}}\left\| {\varepsilon \left( {E_{0} \frac{\partial \psi }{{\partial r}} + \frac{\partial \psi }{{\partial r}}\frac{\partial \psi }{{\partial z}}} \right)} \right\| = 0\,\,\,\,\,\,{\text{at}}\,\,\,\,\,\,\,r = R + \eta \hfill \\ \end{gathered}$$

From the preservation of momentum at zero-order, one obtains:

38 $$\Gamma_{2} - \Gamma_{1} = \left( {\rho_{2} - \rho_{1} } \right)gz + \left( {\zeta_{2} V_{2} /m_{2} - \zeta_{1} V_{1} /m_{1} } \right) - \sigma /R - E_{0}^{2} \left( {\varepsilon_{2} - \varepsilon_{1} } \right)/2 .$$

The nonlinear typical equation which regulates the boundary deflection is attained, by considering that the value of the function $\eta$ is slight. Successively, only the third-order quantities in $\eta$ are calculated.

## Method of solution

To this end, the explanations of the regulating supply functions will be grounded on the standard mode procedure. Following Chandrasekhar^34^, the solution of $\varphi$ and $\psi$ is given as follows:

39 $$\varphi_{j} (r,z;t) = \hat{\varphi }_{j} (r;t)e^{ikz} + c.c.,$$

and

40 $$\psi_{j} (r,z;t) = \hat{\psi }_{j} (r;t)e^{ikz} + c.c.$$

Substituting Eqs. (14), (15), (21) and (22) into the nonlinear border restrictions as provided in Eqs. (23)–(27), and (32)–(34), one realizes that the solutions that rely on the preceding nonlinear border can be represented in the subsequent form:

41 $$\varphi_{1} (r,z;t) = \frac{{m_{1} }}{k}\frac{{\left[ {K_{1} (k\,a)\,I_{0} (k\,r) + K_{0} (k\,r)\,I_{1} (k\,a)} \right]}}{{(\,f_{1} - \,i\,\eta_{z\,} g_{1} )}}\left[ {\frac{\partial \eta }{{\partial t}} + \frac{{V_{1} }}{{m_{1} }}\eta_{z} + \frac{{\alpha_{1} }}{{\rho_{1} }}\left( {\eta + \alpha_{2} \eta^{2} + \alpha_{3} \eta^{3} } \right)} \right],$$

42 $$\varphi_{2} (r,z;t) = \frac{{m_{2} }}{k}\frac{{\left[ {K_{1} (k\,b)\,I_{0} (k\,r) + K_{0} (k\,r)\,I_{1} (k\,b)} \right]}}{{(\,f_{2} - \,i\,\eta_{z\,} g_{2} )}}\left[ {\frac{\partial \eta }{{\partial t}} + \frac{{V_{2} }}{{m_{2} }}\eta_{z} + \frac{{\alpha_{1} }}{{\rho_{1} }}\left( {\eta + \alpha_{2} \eta^{2} + \alpha_{3} \eta^{3} } \right)} \right],$$

43 $$\psi_{1} (r,z;t) = \frac{{\,E_{0} }}{\Delta \,k}\eta_{z} (\varepsilon_{2} - \varepsilon_{1} )(\,g_{2} - \,i\,\eta_{z\,} f_{2} )\left[ {K_{1} (k\,a)\,I_{0} (k\,r) + K_{0} (k\,r)\,I_{1} (k\,a)} \right]\,,$$

and

44 $$\psi_{2} (r,z;t) = \frac{{\,E_{0} }}{\Delta \,k}\eta_{z} (\varepsilon_{2} - \varepsilon_{1} )(\,g_{1} - \,i\,\eta_{z\,} f_{1} )\left[ {K_{1} (k\,b)\,I_{0} (k\,r) + K_{0} (k\,r)\,I_{1} (k\,b)} \right]\,,$$

where $$\Delta = - \,\varepsilon_{2} \,g_{1} \,f_{2} \, + \varepsilon_{1} \,g_{2} \,f_{1} + i\,\eta_{z} \,(\varepsilon_{2} - \varepsilon_{1} )(\,f_{1} \,f_{2} + \,g_{1} \,g_{2} ) + \eta_{z}^{2} (\varepsilon_{2} f_{1} \,g_{2} - \varepsilon_{1} \,g_{1} \,f_{2} )\,,$$ $$f_{1} \, = K_{1} (k\,a)\,I_{1} (k\,R) - K_{1} (k\,R)\,I_{1} (k\,a)\,,\,\,f_{2} \, = K_{1} (k\,b)\,I_{1} (k\,R) - K_{1} (k\,R)\,I_{1} (k\,b)\,,$$ $$g_{1} \, = K_{1} (k\,a)\,I_{0} (k\,R) + K_{0} (k\,R)\,I_{1} (k\,a)\,$$ and $$g_{2} \, = K_{1} (k\,b)\,I_{0} (k\,R) + K_{0} (k\,R)\,I_{1} (k\,b)\,.$$

The obtained distributions of the potential functions $\varphi$ and $\psi$ involve nonlinear terms. As stated before, this nonlinearity takes place in agreement with the inclusion of the nonlinear border restrictions which are previously reported. Ignoring the nonlinear terms, the linear profile arises and is equivalent to those obtained earlier by Chandrasekhar^34^ and Melcher^35^.

In the typical principal stresses that are given in Eqs. (41)–(44), Eq. (37) will be used. This nonlinear characteristic equation can be used in both a linear and nonlinear way to evaluate the stability of the present study. The boundary value problem provides nonlinear solutions, which are provided in Eqs. (41)–(44). As stated in Eq. (37), these solutions will be substituted into the typical stress tensor. The quantity of surface tension determines how dramatically this equation jumps during the interface. This equation is a complex second-order differential equation that is nonlinear. Consequently, the nonlinear characteristic equation is:

45 $$\begin{gathered} c_{1} \eta_{tt} + \left( {c_{2} + id_{1} } \right)\,\eta_{t} + c_{3} \,\eta_{zt} + \left( {c_{4} + id_{2} } \right)\,\eta - \sigma \,\eta_{zz} + [c_{5} + i\,\,(d_{31} \, + d_{32} \,E_{0}^{2} )]\,\eta_{z} + c_{6} \eta_{t}^{2} \hfill \\ + \left( {c_{7} + id_{4} } \right)\,\eta^{2} + [(c_{81} \, + c_{82} \,E_{0}^{2} ) + i\,d_{5} ]\,\eta_{z}^{2} + \left( {c_{9} + id_{6} } \right)\,\eta \eta_{z} + \left( {c_{10} + id_{7} } \right)\,\eta_{t} \eta_{z} + c_{11} \,\eta_{t} \eta \hfill \\ + i\,d_{8} \,\eta_{zt} \eta_{z} + i\,d_{9} \,\eta_{tt} \eta_{z} + c_{12} \,\eta_{tt} \eta_{z}^{2} + c_{13} \,\eta_{t} \eta^{2} + c_{14} \,\eta_{zt} \eta_{z}^{2} + \left( {c_{15} + id_{10} } \right)\,\eta_{t} \eta_{z}^{2} + i\,d_{11} \,\eta_{z} \eta_{t} \,\eta \hfill \\ + i\,d_{12} \,\eta_{t}^{2} \,\eta_{z} + \frac{\sigma }{2}\eta_{zz} \,\eta_{z}^{2} + \left( {c_{16} + id_{13} } \right)\,\eta^{3} + [c_{17} + i\,(d_{141} \, + d_{142} \,E_{0}^{2} )]\,\eta_{z}^{3} + \left( {c_{18} + id_{15} } \right)\,\eta \eta_{z}^{2} = 0 \hfill \\ \end{gathered}$$

where the constants $c_{i}$, $i = 1,2,...,18$ and $d_{j}$,$j = 1,2,...,15$ are listed in the Appendix.

## Standard stability analysis

To explore the linear stability examination, imagine a constant monochromatic wave train solution as:

46 $$\eta (r,z;t) = \Lambda \,e^{i(kz - wt)} ,$$

where $\Lambda$ is a little parameter which controls the performance of the amount of the interface disruption. In the following linear approach, the amplitude $\Lambda$ may be taken as a small constant.

Now, by introducing Eq. (46) into Eq. (45), this process generates an extremely difficult nonlinear equation in $\eta$. Suppose that the displacement function $\eta$ is little. The nonlinear equation of the interface displacement is calculated up to the third order of $\eta$. Therefore, the nonlinear characteristic formula in terms of the interfacial displacement $\eta$ can be expressed as:

47 $$F(\omega ,\,k)\eta = H(\omega ,\,k)\,\eta^{2} + \Theta (\omega ,\,k)\,\eta^{3} ,$$

In the linear stability methodology, the linear expression of the nonlinear dispersion relationship (47) is introduced by omitting the higher powers of $\eta$, to attain the equation:

48 $$F(\omega ,\,k)\eta = 0.$$

Employing Eq. (46) into Eq. (48) and since $\eta$ should be of a non-zero value, it is found that

49 $$s_{0} \omega^{2} + (s_{1} + ib_{1} )\omega + s_{2} + ib_{2} = 0 ,$$

where the coefficients $s_{0} ,\,s_{1} ,\,b_{1} ,\,s_{2} ,\,\,\,{\text{and}}\,\,\,\,b_{2}$ are moved to the Appendix.

Equation (49) is a linear dispersion expression that is represented by the two cylindrical liquids with the central axis. It is made up of the wave number as well as the growth rate that correspond**s** to constraints. Via the linear stability approach, the conduct of growth rate controls whether the duration of time is stable or unstable. If the imaginary portion of $\omega$ is positive, the instability will eventually spread. So, the mechanism develops linear instability. The mechanism would be linearly stable when the imaginary component is negative since it will lead to a decrease in the disturbance. So, the stability or instability is conditional on how the real component of the mechanism behaves and whether it oscillates for any period of time. The main focus of this section is on investigating the mechanism stability via a linear methodology.

The dispersion relationship (50) determines the stability criteria that are produced using the Routh-Hurwitz theory^36^. In a few words, these criteria necessitate that the imaginary portion be negative:

50 $$s_{0} > 0,\,\,\,\,\,\,b_{1} > 0,\,\,\,\,\,and\,\,\,\,\,\,\,s_{1} b_{1} b_{2} - s_{0} b_{2}^{2} - s_{2} b_{1}^{2} > 0$$

Actually, the first two requirements of restrictions in (50) should be considered when dealing with the stability image. Simultaneously, the interpretation of the third condition reveals that it relies on $E_{0}^{2}$. Consequently, this is the main condition of stability. Consequently, the electric strength $E_{0}^{2}$ will be drawn against the wave number of the surface wave $k$. In reality, the first two conditions (50) must be satisfied. The mathematical formulations of the first two conditions showed that these conditions are independent of the electric field intensity $E_{0}^{2}$. Subsequently, the preceding computations are done, so the domain, in the phase plane, should meet these conditions automatically. The third Inequality of Eq. (50) is changed to the following condition:

51 $$\,\,\,E_{0}^{2} > \frac{1}{{s{}_{22}b_{1}^{2} }}\left( {s_{0} b_{2}^{2} + s_{21} b_{1}^{2} - s_{1} b_{1} b_{2} } \right) .$$

Before performing the numerical computations, for more appropriateness, Eq. (51) should be expressed in a satisfactory dimensionless structure. This is done in a set of methods relying mainly on the selection of the following properties: $\sqrt {\rho R^{3} /\sigma }$, $R$, and $\rho R^{3}$ are time, length, and mass, correspondingly. The other dimensionless amounts can be signified as follows: $\rho^{*} = \rho_{2} /\rho_{1}$ is the proportion of fluid densities, $\gamma^{*} = \gamma_{2} /\gamma_{1}$ is the proportion of limiting viscosities, $\alpha = \alpha_{1} /\tilde{\alpha }_{1}$ is the ratio of the liner MHT coefficient, $\varepsilon^{*} = \varepsilon_{2} /\varepsilon_{1}$ is the proportion of dielectric constants, $V^{*} = V_{2} /V_{1}$ is the ratio of speeds, $m^{*} = m_{2} /m_{1}$ is the ratio of the porosity factors, $E_{0}^{*2} = E_{0}^{2} R\,\varepsilon_{1} /\sigma$ is a dimensionless electric strength, $We = \rho_{1} \,V_{1}^{2} R/\sigma$ is the liquid Weber number, $Da = \lambda /R^{2}$ is the Darcy number, and $Z = \gamma_{1} /\sqrt {\rho_{1} \,V_{1} \,R}$ is Ohnesorge number.

**Figure 2:**
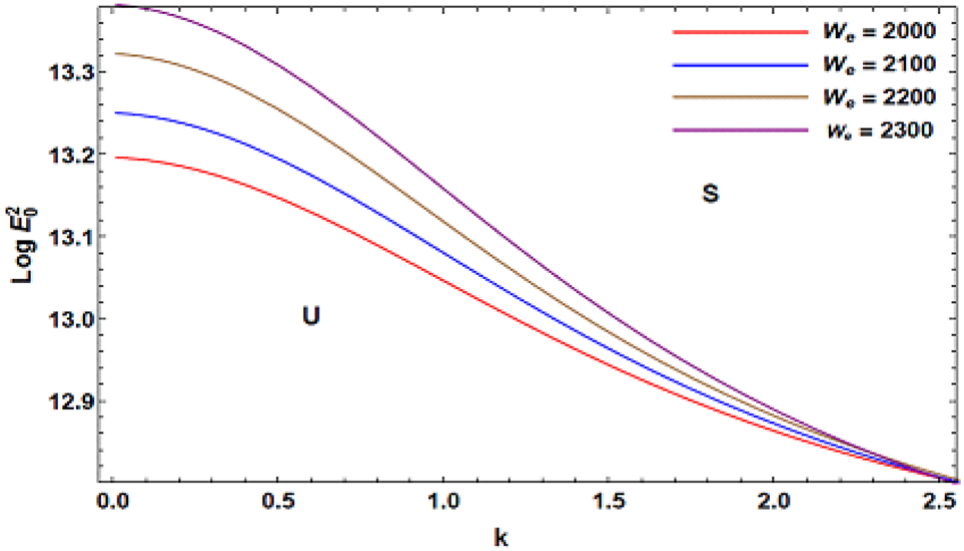
linear stability shape corresponding to Eq. (51) for the deviation of $We$.

**Figure 3:**
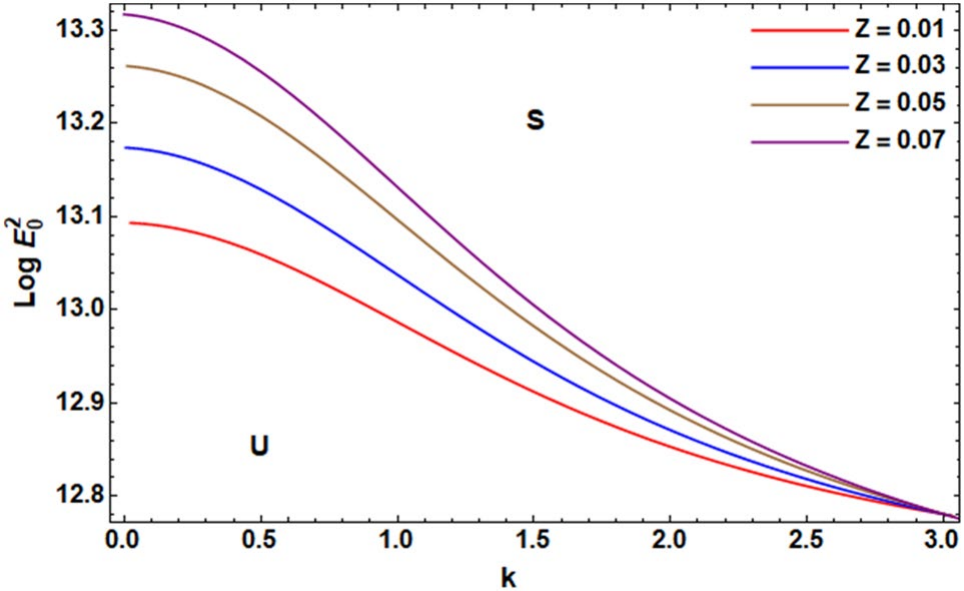
Linear stability profile corresponding to Eq. (51) for the deviation of $Z$.

**Figure 4:**
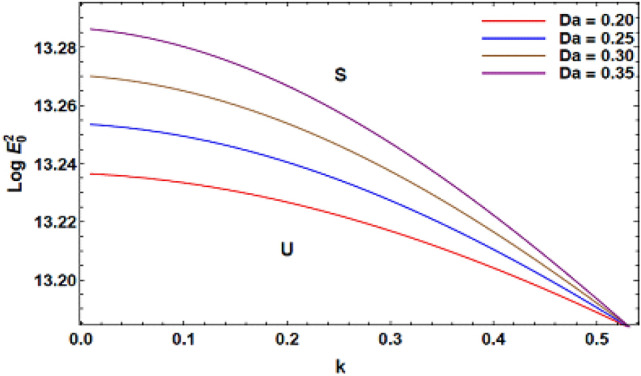
Linear stability shape corresponding to Eq. (51) for the deviation of $Da$.

**Figure 5:**
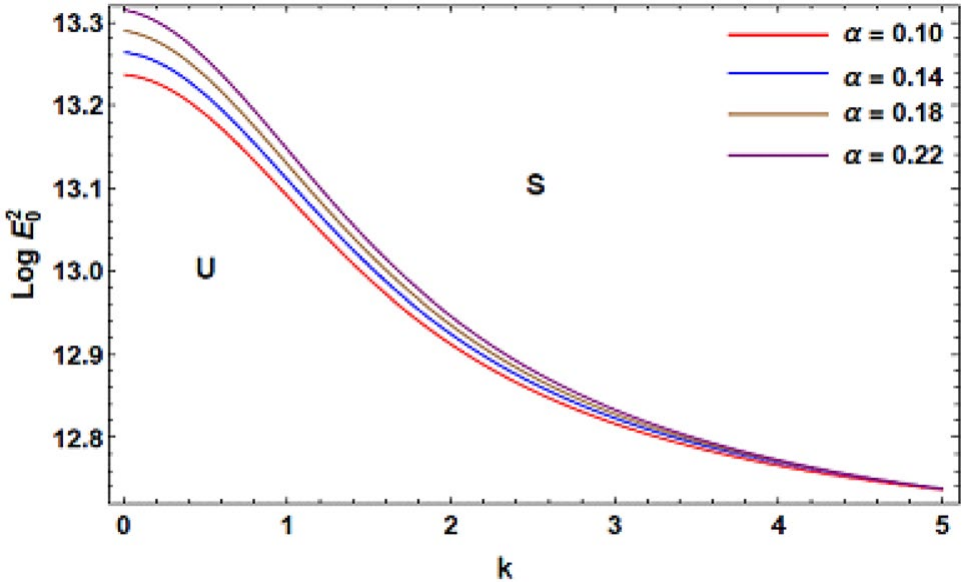
Linear stability shape corresponding to Eq. (51) for the deviation of $\alpha$.

For ease of follow-up, the mark ($*$) can be disregarded in the subsequent estimation. Hereafter, a numerical calculation would be sketched for the stability standard (51) for the deviation of the electric strength $E_{0}^{2}$ against the wave number $k$. The aim of this analysis is to indicate the stabilizing impact of various factors of the model. These parameters are given as the Weber number $We$, Ohnesorge number $Z$, Darcy number $Da$ and the MHT coefficient $\alpha$. In the following figures, the symbol S indicates the stable regions, while the symbol U symbolizes the unstable ones. Corresponding to the stability standard (51), the zone upper the curve signifies the stable zone, while the zone under the curve symbolizes the unstable one. Really, this demonstrates a stabilizing impact of the longitudinal electric strength. Numerous sources were given, as well as the nonlinear stability methodology, to support this conclusion. Moreover, Figs. 2, 3, 4 and 5 have the specific values:

$$\rho = 1,\,a = 0.2,\,\,b = 0.3,\,\,\varepsilon = 0.8,\,\,V = 0.3,\,Z = 0.09,\,\,Da = 0.2,\,\,We = 2000,\,\xi = 0.8,\,\,\tilde{\alpha }_{1} = 0.1,\,\,\gamma = 0.9,\,\,m_{1} = 0.3\,\,{\text{and}}\,\,m_{2} = 0.2.$$

Figure 2 depicts the deviation of $Log\,E_{0}^{2}$ against the dimensionless wave number $k$ for various amounts of Weber number $\,We$. Physically, Weber number $\,We$ is regularly helpful in examining liquid streams, where there exists a boundary connecting two distinct liquids, particularly for multi-phase streams as well as sharply arched surfaces. This Weber numeral determines the proportion of the momentum due to the vapor layer when separated by the amount of the surface tension that restricts the fluid. This graph illustrates how the unstable zone increases as the values $\,We$ is enhanced. Consequently, the Weber numeral causes the technique within consideration to become unstable. This demonstrates that the non-Newtonian liquid cylinders become unstable when their velocity, radius, and density are amplified. At higher liquid Weber numerals, it is revealed that the non-Newtonian fluid cylinder destabilizes more readily. Recently, similar results have been verified^7^.

Figure 3 shows the deviation of $Log\,E_{0}^{2}$ versus $k$ for distinct amounts of Ohnesorge number $Z$. Ohnesorge number is concerned with the forces of viscosity as well as surface tension. Regularly, it is utilized in free surface liquid mechanisms like dispersion of fluids in gases as well as in spray equipment. It is depicted from this figure that as Ohnesorge number develops (or Reynolds numeral is decayed), the instability region decreases. Significantly, at the higher Ohnesorge number, the internal viscous dissipation becomes more controlling. This implies that most of the introduced energy change**s** into internal viscous dissipation. This decreases the velocity of the liquid. Consequently, the stability regions increase. By contrast, at the lower value of the Ohnesorge number, the friction becomes weaker during viscous forces. Therefore, the stability regions decrease. Henceforth, one can deduce that the Ohnesorge numeral has a destabilizing impact on the considered structure. Similar outcomes were found in Refs.^7,37^.

Figure 4 illustrates the variation of $Log\,E_{0}^{2}$ against $k$ for several amounts of Darcy factor $Da$, which is recognized as non-dimensional medium permeability. The Darcy number has a vital function in heat transmission through permeable substances such as metals. In fluid dynamics due to permeable surrounding, the Darcy number indicates the relative impact of the permeability of the milieu against its cross-sectional zone, generally, when the diameter is squared. As shown by numerous investigators, the rise in the cylinder radius has a stabilizing effect. Correspondingly, the Darcy number is inversely proportional to the square of the radius. Therefore, it leads to a destabilizing influence on the investigated mechanism. This result agrees with earlier outcomes^38^. Consequently, the temperature gradient due to the hot wall rises with the rise of the Darcy number. Thereafter, speed of the mechanism improves and exerts a destabilizing impact. A Similar result was previously found^39^.

Figure 5 displays the stability shape in $Log\,E_{0}^{2} - k$ plane at various amounts of the MHT parameter $\alpha_{1}$. As observed from the mathematical definition of this parameter, the MHT coefficient is inversely proportional to both the jet radius and the latent heat $L$ that is transformed from one fluid to the other. Physically, the latent heat is recognized as latent heat of conversion. It is defined as the energy disseminated, by a body, due to a regular-heat procedure. It is realized from this figure that the stable zone diminishes as $\alpha_{1}$ rises. Consequently, it is shown that MHT has a destabilizing impact. Physically, as MHT improves through the interface, additional temperature conveyed to a point at less heat is eliminated from the position. A similar result was confirmed in Refs.^40–42^.

## Nonlinear stability investigation

To discuss the nonlinear stability of the procedure under consideration, the scheme is improved to merge the nonlinear terms and the linear dispersion relationship $F(\omega ,k) = 0$, which is a modification of the wave train. The process of multiple scales method was provided^43^. The primary concept of this procedure is to manage an extension, indicating a procedure of solution, as a function of two or more independent factors. Assume a little non-dimensional factor $\delta$ which computes the proportion of a typical wavelength or time scale of the improvement. These factors $x$ and $t$, which are computed on scales of an ideal wavelength and periodic time, may be extended to alternate factors as:

52 $$T_{n} = \delta^{n} t\,\,{\text{and}}\,\,X_{n} = \delta^{n} x,\,\,n = 1,2, \ldots$$

Thus, the operators can be designated as:

53 $$\frac{\partial }{\partial t} \equiv - \omega \frac{\partial }{{\partial \theta_{0} }} + \delta \frac{\partial }{{\partial T_{1} }} + \delta^{2} \frac{\partial }{{\partial T_{2} }} + ....,$$

54 $$\frac{\partial }{\partial x} \equiv - k\frac{\partial }{{\partial \theta_{0} }} + \delta \frac{\partial }{{\partial X_{1} }} + \delta^{2} \frac{\partial }{{\partial X_{2} }} + ....,$$

55 $$\frac{{\partial^{2} }}{{\partial t^{2} }} \equiv \omega^{2} \frac{{\partial^{2} }}{{\partial \theta_{0}^{2} }} - 2\omega \delta \frac{{\partial^{2} }}{{\partial \theta_{0} \partial T_{1} }} + \delta^{2} \left( {\frac{{\partial^{2} }}{{\partial T_{1}^{2} }} - 2\omega \frac{{\partial^{2} }}{{\partial T_{2} \partial \theta_{0} }}} \right) + ....,$$

and

56 $$\frac{{\partial^{2} }}{{\partial x^{2} }} \equiv k^{2} \frac{{\partial^{2} }}{{\partial \theta_{0}^{2} }} + 2k\delta \frac{{\partial^{2} }}{{\partial \theta_{0} \partial X_{1} }} + \delta^{2} \left( {\frac{{\partial^{2} }}{{\partial X_{1}^{2} }} + 2k\frac{{\partial^{2} }}{{\partial X_{2} \partial \theta_{0} }}} \right) + .....,$$

where $$\theta_{0} = kX_{0} - \omega T_{0}$$.

The evaluation concerned with a perturbation mechanism and repression of the secular terms was exhibited^44^. This procedure leads to the Ginzburg–Landau formula. Utilizing the same manner as previously shown^44^, the Ginzburg–Landau formula takes the following structure:

57 $$i\frac{\partial \Lambda }{{\partial \Xi }} + \left( {P_{r} + iP_{i} } \right)\frac{{\partial^{2} \Lambda }}{{\partial \zeta^{2} }} = \left( {Q_{r} + iQ} \right)\Lambda^{2} \overline{\Lambda },$$

where $\overline{\Lambda }$ denotes the complex conjugate of $\Lambda$ and

58 $$\begin{aligned} P_{r} + iP_{i} &= - \frac{1}{2}\left( {V_{g}^{2} \frac{{\partial^{2} F}}{{\partial \omega^{2} }} + 2V_{g} \frac{{\partial^{2} F}}{\partial \omega \partial k} + \frac{{\partial^{2} F}}{{\partial k^{2} }}} \right)\left( {\frac{\partial F}{{\partial \omega }}} \right)^{ - 1} , \nonumber \\ Q_{r} + iQ_{i} &= \left( {\frac{{2H^{2} }}{\Omega } + \Theta } \right)\left( {\frac{\partial F}{{\partial \omega }}} \right)^{ - 1} , \nonumber\\ \zeta & = (x - V_{g} t)\delta ,\,\,\,\,\Xi = \delta^{2} t.\end{aligned}$$

The group speed $V_{g}$ is specified by $V_{g} = - \frac{\partial F}{{\partial k}}\left( {\frac{\partial F}{{\partial \omega }}} \right)^{ - 1}$, $\Omega = F(2\omega ,2k)$.

Obviously, the values of amounts $P$ as well as $Q$ are computed from the performance of the parameters of Eq. (47).

The stability gauges of the Ginzburg–Landau Eq. (57) have been illustrated^45^. The stability criteria may be presented as:

59 $$Q_{i} < 0\,\,{\text{and}}\,\,P_{r} Q_{r} + P_{i} Q_{i} > 0.$$

The transition curves, detaching the stable and unstable zones, are detected by

60 $$Q_{i} = 0\,\,{\text{and}}\,\,P_{r} Q_{r} + P_{i} Q_{i} = 0.$$

The non-dimensional formula counts on the selection of length, time, and mass, which take the following amounts:

Length is represented by $g/\omega^{2}$, time stands for $1/\omega$ and mass is recognized as $\sigma /\omega^{2}$. The other dimensionless quantities can be formulated as:

61 $$\begin{aligned} & k = \frac{{k^{*} \omega^{2} }}{g},\,\,\rho_{1} = \rho^{*} \rho_{2} ,\,\,V_{1} = V^{*} V_{2} ,\,\,\nu_{1} = \nu^{*} \nu_{2} ,\,\,\xi_{1} = \xi^{*} \xi_{2} ,\\ & E^{2} = \frac{{E^{*2} \omega^{2} \sigma }}{g} \quad \alpha_{1} = \frac{{\alpha_{1}^{*} \sigma \omega^{5} }}{{g^{3} }},\,\,\alpha_{2} = \frac{{\alpha_{2}^{*} \omega^{2} }}{g}\,\,\,{\text{and }}\,\alpha_{3} = \frac{{\alpha_{3}^{*} \omega^{4} }}{{g^{2} }}. \end{aligned}$$

where the superstar stands for the dimensionless amounts.

In view of linear stability theory, the stability standard is evaluated by one transition curve. Thereafter, the stability chart is detached by only one curve. Meantime, throughout the nonlinear stability mechanism, there are several transition curves separating the stability profile into stable or unstable parts. Thence, the nonlinear stability mechanism is more accurate than the linear one.

Currently, we consider the stability diagram throughout the nonlinear mechanism. The curves specifying the stipulation $Q_{i} = 0$ are reformulated as an equation of third order of $E_{0}^{2}$ as follows:

62 $$M_{1} \left( {E_{0}^{2} } \right)^{3} + M_{2} \left( {E_{0}^{2} } \right)^{2} + M_{3} \left( {E_{0}^{2} } \right) + M_{4} = 0.$$

Meantime, the stability circumstance $P_{r} Q_{r} + P_{i} Q_{i} = 0$ is designated as an equation of fifth order of $E_{0}^{2}$ as:

63 $$N_{1} \left( {E_{0}^{2} } \right)^{5} + N_{2} \left( {E_{0}^{2} } \right)^{4} + N_{3} \left( {E_{0}^{2} } \right)^{3} + N_{4} \left( {E_{0}^{2} } \right)^{2} + N_{5} \left( {E_{0}^{2} } \right) + N_{6} = 0,$$

where $M^{\prime}s$ and $N^{\prime}s$ rely on all factors of the procedure and are listed in the Appendix.

**Figure 6:**
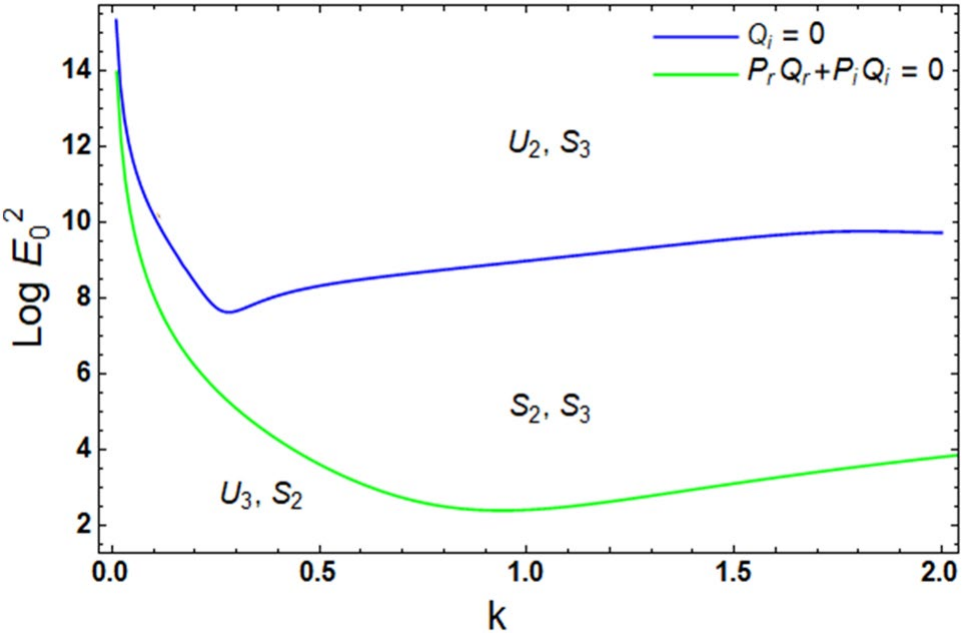
Exhibits the contribution of the two stipulations (62) and (63).

During the nonlinear numerical evaluation, $\log E_{0}^{2}$ is plotted in contrast to $k$. The two Eqs. (62) and (63) have an odd order. Thence, at least, one real root is achieved for all of them. Indeed, the approved root must be real and positive for all real amounts of $k$. It should be noted that the blue curve refers to the cubic Eq. (62). In the interim, the green curve stands for Eq. (63). The numerical estimation exhibits that Eq. (62) grants three real roots. One of them is positive for the provided scope of $k$. Thus, one curve is only regarded. Furthermore, Eq. (63) generates only one real positive root. The other roots may be either negative or complex. Thence, only one curve is scrutinized. Through the nonlinear stability procedure, it is viewed that the transition curve, which is specified by Eq. (62), detaches the zone of stability into two parts. The first zone is stable ($S_{2}$) and is located under the blue curve. In the interim, the other one is unstable ($U_{2}$) and exists upper this curve. Actually, the new zone of instability is granted by the nonlinear mechanism. Besides, the green curve, which is determined by Eq. (63), separates the zone under the blue curve into an** u**nstable zone ($U_{3}$) under the green curve as well as a stable one ($S_{3}$) upper this curve. It is clear from Fig. 6 that the stable zone lies only between the two curves, otherwise the system becomes unstable.

**Figure 7:**
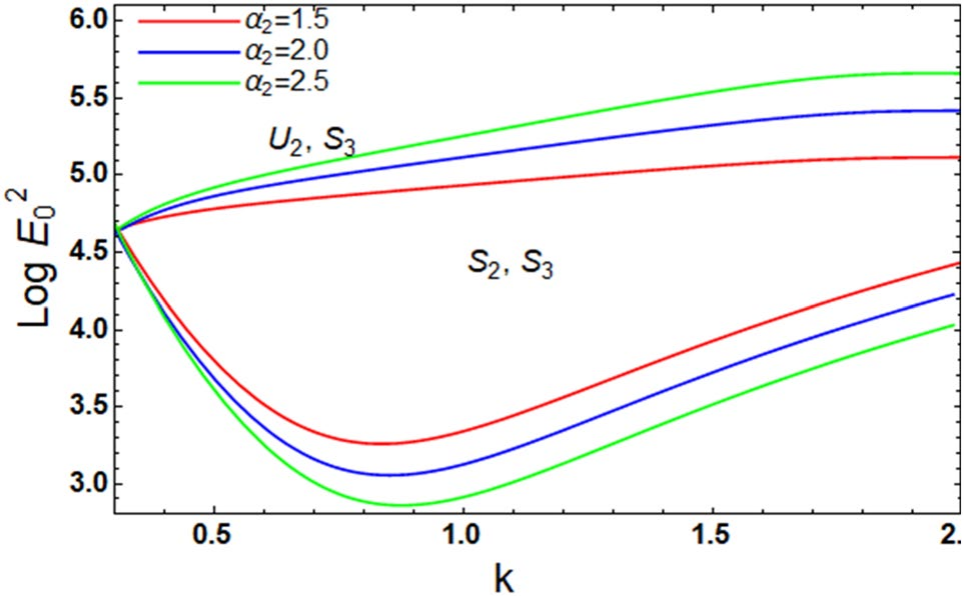
Presents the alteration in $\alpha_{2}$ of inequality (62) and (63).

**Figure 8:**
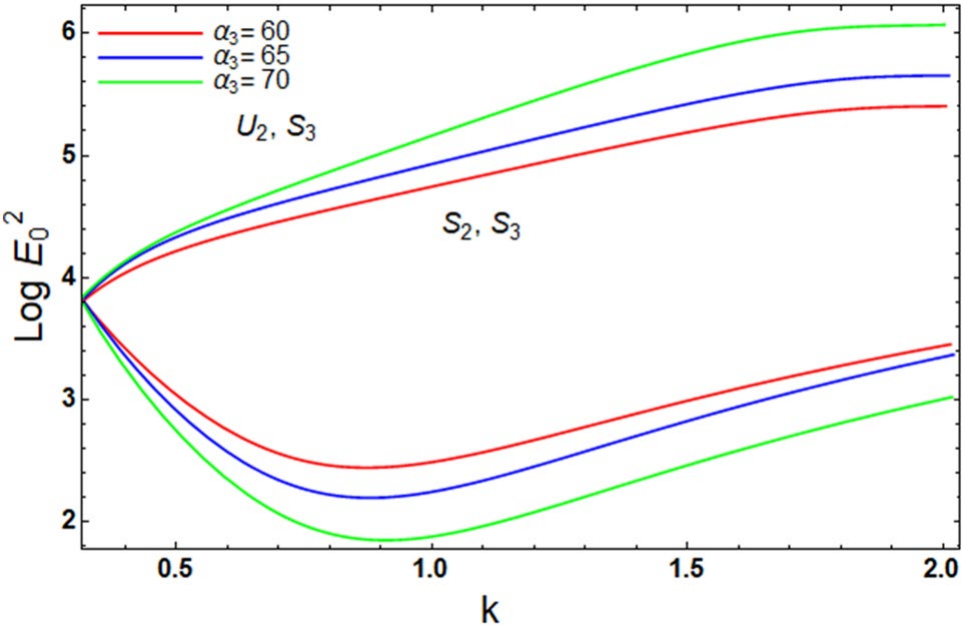
Displays the alteration in $\alpha_{3}$ of inequality (62) and (63).

We are forced to tackle the nonlinear stipulation as specified by Eqs. (62) and (63). Hence, Figs. 7 and 8 stand for the impact of the nonlinear formulas of HMT parameters $\alpha_{2}$ and $\alpha_{3}$. This figure exhibits a stabilizing impact. From a physical standpoint, MHT indicates the net gaging of mass. Indeed, heat transmission impacts concern immediately with the electric influences. The impacts of mass as well as heat transfer are detected within the permeable catalyst particles. This is in distinction with the factor $\alpha_{1}$.

**Figure 9:**
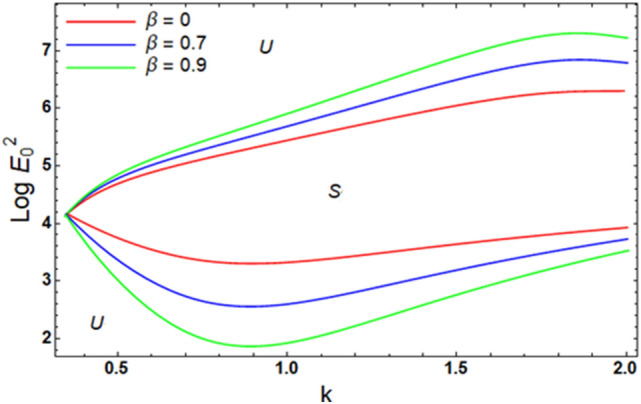
Exhibits the alteration in $\beta$ of inequality (62) and (63).

Figure 9 identifies the impact of cross viscosity $\beta$ on the stability diagram. It is recognized that the proportion $\beta$ has a stabilizing impact. The absence of the proportion factor $\beta$ means that the liquid turns into a Newtonian one. Nevertheless, $\beta \ne 0$ implies that the liquid converts into the non-Newtonian manner. Thence, the non-Newtonian conduct is more stabilizing than the Newtonian one. It must be understood^38^ that the stability procedure of the Rivlin-Ericksen type flows in the inviscid gas. They observed a similar conduct of $\beta$. Furthermore, the current outcomes, concerning the cross viscosity, exhibit certain uniformity with the previously published findings.

**Figure 10:**
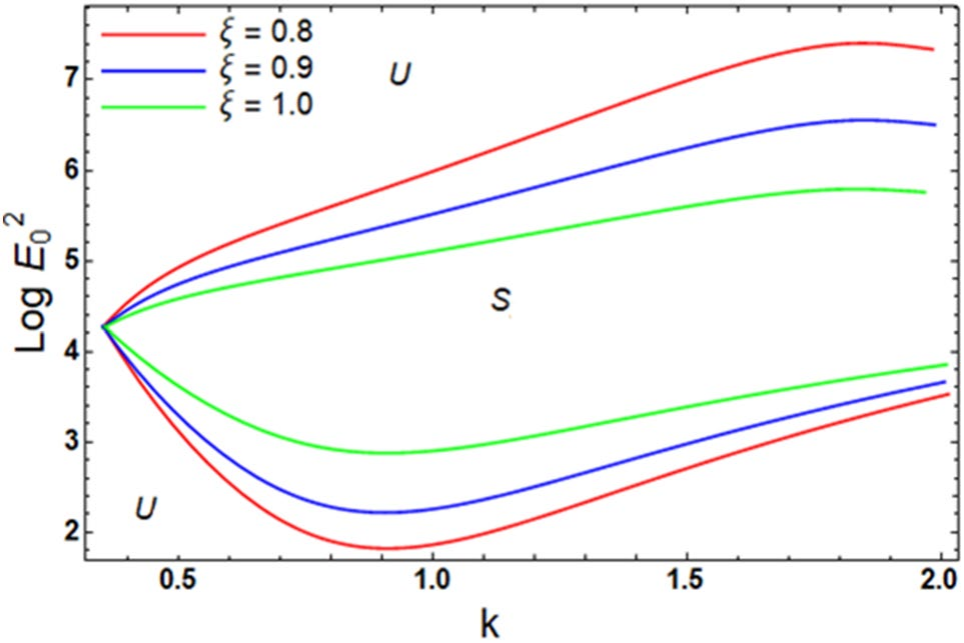
Exhibits the alteration in $\xi$ inequality (62) and (63).

The presence of the porous scheme with varied amounts of the proportion of the Darcy’s factor $\xi$ in the stability diagram is scrutinized via Fig. 10. As elucidated from this figure, the improvement in the proportion $\xi$ grants a boost in the instability district. Thence, $\xi$ brings about a destabilizing impact. From the physical standpoint, this takes place throughout the drag force. This outcome is in line with what has already been discovered.

**Figure 11:**
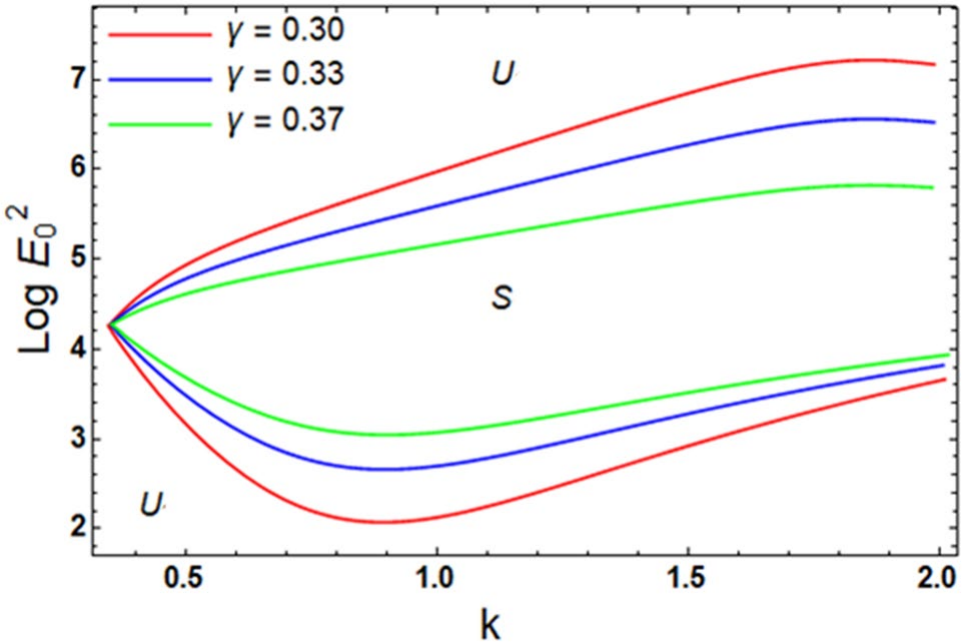
Exhibits the alteration in $\gamma$ of inequality (62) and (63).

Figure 11 exhibits the stability structure of diverse amounts of the linear proportion viscosity $\gamma$. It is displayed that the stable zone is diminished by the rise in as $\gamma$. Thence, it is elucidated that $\gamma$ has a destabilizing impact. Actually, $\gamma$ leads to improving the resistance of the liquid. Consequently, the speed of the movement is reduced. It is recognized that the viscosity grants resistance to movement. Further, the present outcomes are consistent with our previous findings^7,46^.

**Figure 12:**
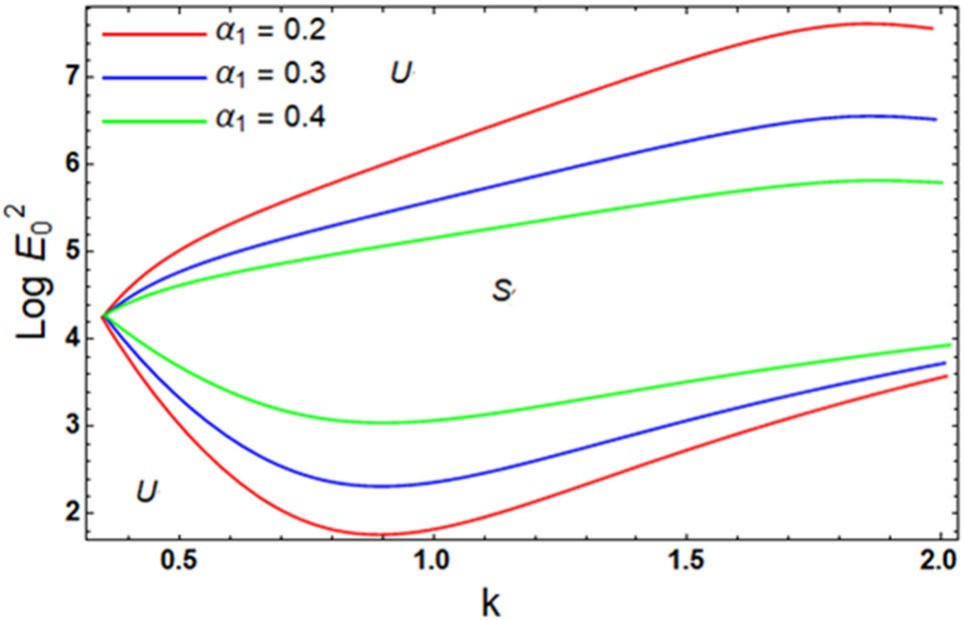
Exhibits the alteration in $\alpha_{1}$ of inequality (62) and (63).

Figure 12 examines the stability scheme for distinct amounts of heat transference factor $\alpha_{1}$. It is indicated that the stable zone is diminished by the increase of $\alpha_{1}$. Thence, it is exhibited that the transfer of heat as well as mass transfer brings about a destabilizing impact. The increase in MHT through the boundary can transmit further heat.

## The surface displacement profile

As mentioned in the preceding section, substituting from Eq. (46) into Eq. (45) yields the equation governing the nonlinear stability approach. As suggested, it expresse**s** a partial differential formula in a nonlinear form involving complex factors in terms of boundary elevation $\eta {\text{(z, t)}}{.}$ Actually, the examination of this formula, in its existing format, is complicated. Actually, the performance of $\eta {\text{(z, t)}}$ must be real. To simplify the following estimations, one may assume the following initial condition $\eta = \eta (0,t) = \Lambda (t)$. Thence, Eq. (45) can be split into its real and imaginary parts as:

64 $$\Lambda^{\prime\prime} + l_{1} \,\Lambda^{\prime} + l_{2} \,\Lambda + l_{3\,} \,\Lambda^{{\prime}{2}} + l_{4\,} \,\Lambda^{2} + l_{5} \,\Lambda \,\Lambda^{\prime\prime} + l_{6} \,\Lambda \,\Lambda^{\prime} + l_{7} \,\Lambda^{3} + l_{8} \Lambda^{2} \,\Lambda^{\prime\prime} + + l_{9} \,\Lambda^{2} \,\Lambda^{\prime} + l_{10} \,\Lambda \,\Lambda^{{\prime}{2}} = 0 ,$$

and

65 $$L_{1} \,\Lambda^{\prime} + L_{2} \,\Lambda + L_{3\,} \Lambda^{2} + L_{4} \,\Lambda \,\Lambda^{\prime} + L_{5} \,\Lambda^{3} + L_{6} \,\Lambda^{2} \,\Lambda^{\prime} = 0 ,$$

where $l_{i} ,\,L_{i} \,(i = 1,2,....)$ are obvious given the situation. They are listed in the Appendix.

Thereafter, the incorporation of Eqs. (64) and (65) may be granted by excluding $\Lambda^{\prime}$ among them. The equation may be formulated as:

66 $$\Lambda^{\prime\prime} + \varpi^{2} \,\Lambda + A_{1\,} \Lambda^{2} + A_{2} \,\Lambda \,\Lambda^{\prime\prime} + A_{3} \,\Lambda^{3} + A_{4} \,\Lambda^{2} \,\Lambda^{\prime\prime} = 0.$$

where $\varpi^{2} ,A_{i} ,\,(i = 1,2,....)$ represents the natural frequency of the system. Therefore, it should be positive. They are moved to the Appendix.

Currently, the amplitude of Eq. (50) grants real coefficients. It stands for a simplified cubic nonlinear differential formula. To tackle this equation, it demand**s** preliminary requirements. For this objective, the following initial criteria are included:

67 $$\Lambda \left( 0 \right) = 0;\,\,\,\,\,\,\,\,{\text{and}}\,\,\,\,\,\,\,\,\Lambda^{\prime}(0) = 1.$$

For this target, the homotopy expression becomes

68 $$\Lambda^{\prime\prime} + \varpi^{2} \,\Lambda + \delta \left( {A_{1\,} \Lambda^{2} + A_{2} \,\Lambda \,\Lambda^{\prime\prime} + A_{3} \,\Lambda^{3} + A_{4} \,\Lambda^{2} \,\Lambda^{\prime\prime}} \right) = 0;\,\,\delta \in \left[ {0,\,1} \right].$$

This study will rely on extending the expanded frequency manner. In agreement with this procedure, suppose a nonlinear frequency ${\rm X}^{2}$, so it can be extended as:

69 $${\rm X}^{2} = \varpi^{2} + \sum\limits_{j = 1}^{\infty } {\delta^{j} \varpi \, _{j} } .$$

Merging Eqs. (68) and (69), one gets

70 $$\Lambda^{\prime\prime} + {\rm X}^{2} \,\Lambda + \delta \left( { - (\varpi_{1} + \delta \,\varpi_{2} )\Lambda + A_{1\,} \Lambda^{2} + A_{2} \,\Lambda \,\Lambda^{\prime\prime} + A_{3} \,\Lambda^{3} + A_{4} \,\Lambda^{2} \Lambda^{\prime\prime}} \right) = 0.$$

Considering Laplace transforms to both sides of Eq. (70), and considering the initial stipulations (67), the outcome becomes

71 $$L_{T} \left\{ {\Lambda (t;\,\delta )} \right\} = \frac{S}{{S^{2} + {\rm X}^{2} }} - \frac{1}{{S^{2} + {\rm X}^{2} }}L_{T} \left\{ {\delta \left( { - (\varpi_{1} + \delta \,\varpi_{2} )\Lambda + A_{1\,} \Lambda^{2} + A_{2} \,\Lambda \,\Lambda^{\prime\prime} + A_{3} \,\Lambda^{3} + A_{4} \,\Lambda^{2} \,\Lambda^{\prime\prime}} \right)} \right\}\,.$$

Utilizing the inverse transform of both sides of Eq. (71), one realizes

72 $$\Lambda (t;\,\delta ) = L_{T}^{ - 1} \left( {\frac{S}{{S^{2} + {\rm X}^{2} }}} \right) - L_{T}^{ - 1} \left[ {\frac{1}{{S^{2} + \sigma^{2} }}L_{T} \left\{ {\delta \left( { - (\varpi_{1} + \delta \,\varpi_{2} )\Lambda + A_{1\,} \Lambda^{2} + A_{2} \,\Lambda \,\Lambda^{\prime\prime} + A_{3} \,\Lambda^{3} + A_{4} \,\Lambda^{2} \,\Lambda^{\prime\prime}} \right)} \right\}} \right].$$

In agreement with the usual HPM, $\gamma (t;\delta )$ can be extended as:

73 $$\Lambda (t;\delta ) = \sum\limits_{n = 0}^{\infty } {\delta^{n} \Lambda_{n} (t)} = \Lambda_{0} (t) + \delta \Lambda_{1} (t) + \delta^{2} \Lambda_{2} (t) +\cdots$$

Actually, comparing the coefficients of $\Lambda (t;\delta )$ as provided in Eq. (73), then equating the indicial powers $\delta$ on both sides, one finds

74 $$\delta^{0} :\,\Lambda_{0} (t) = \frac{1}{\rm X}\sin {\rm X}\,t\,\,,$$

75 $$\delta \,\,:\,\,\,\Lambda_{1} (t) = - L_{T}^{ - 1} \left[ {\frac{1}{{S^{2} + {\rm X}^{2} }}L_{T} \left\{ { - \varpi_{1} \Lambda_{0} + A_{1\,} \Lambda_{0}^{2} + A_{2} \,\Lambda_{0} \,\Lambda^{\prime\prime}_{0} + A_{3} \,\Lambda_{0}^{3} + A_{4} \,\Lambda_{0}^{2} \,\Lambda^{\prime\prime}_{0} } \right\}} \right] ,$$

76 $$\delta^{2} \,\,:\,\,\Lambda_{2} (t) = - L_{T}^{ - 1} \left[ {\frac{1}{{s^{2} + {\rm X}^{2} }}L_{T} \left\{ { - \varpi_{2} \Lambda_{0} - \varpi_{1} \Lambda_{1} + 2A_{1\,} \Lambda_{0} \Lambda_{1} + A_{2} \,(\Lambda_{0} \,\Lambda^{\prime\prime}_{1} + \Lambda_{1} \,\Lambda^{\prime\prime}_{0} ) + 3A_{3} \,\Lambda_{0}^{2} \Lambda_{1} + A_{4} \,(\Lambda_{0}^{2} \,\Lambda^{\prime\prime}_{1} + 2\Lambda_{0} \Lambda_{1} \,\Lambda^{\prime\prime}_{0} )} \right\}} \right].\,$$

and

77 $$\delta^{3} \,\,:\,\,\,\Lambda_{3} (t) = - L_{T}^{ - 1} \left[ {\frac{1}{{s^{2} + {\rm X}^{2} }}L_{T} \left\{ \begin{gathered} - \varpi_{1} \Lambda_{2} - \varpi_{2} \Lambda_{1} - \varpi_{3} \Lambda_{0} + A_{1\,} (2\Lambda_{0} \Lambda_{2} + \Lambda_{1}^{2} ) + A_{2} \,(\Lambda_{0} \,\Lambda^{\prime\prime}_{2} + \Lambda_{2} \,\Lambda^{\prime\prime}_{0} + \Lambda_{1} \,\Lambda^{\prime\prime}_{1} ) \hfill \\ + 3A_{3} \,(\Lambda_{1}^{2} \Lambda_{0} + \Lambda_{0}^{2} \Lambda_{2} ) + A_{4} \,(\Lambda_{0}^{2} \,\Lambda^{\prime\prime}_{2} + \,\Lambda^{\prime\prime}_{0} (\Lambda_{1}^{2} + 2\Lambda_{0} \Lambda_{2} + 2\Lambda_{0} \Lambda_{1} \Lambda^{\prime\prime}_{1} \,)) \hfill \\ \end{gathered} \right\}} \right].\,$$

On substituting from Eq. (74) into Eq. (75), all factors of $cos{\rm X}t$ or $sin{\rm X}t$ in Eq. (75) generate the secular formulas. Thence, with a view to gain a regular applicable expansion of Eq. (75), these secular formulas should be excluded. Consequently, the amount $\varpi_{1}$ is formulated as

78 $$\varpi_{1} = \frac{3\,}{{4{\rm X}^{2} }}\left( {A_{3} - A_{4} {\rm X}^{2} } \right).$$

It follows that the uniform formula of $\Lambda_{1} (t)$ may be expressed as

79 $$\Lambda_{1} (t) = \frac{{A_{2} {\rm X}^{2} - A_{1} }}{{2{\rm X}^{2} }} + \frac{{2\left( {A_{1} - A_{2} {\rm X}^{2} } \right)}}{{3{\rm X}^{4} }}\cos {\rm X}\,t + \frac{{3\left( {A_{3} - A_{4} {\rm X}^{2} } \right)}}{{32{\rm X}^{5} }}\sin {\rm X}\,t + \frac{{ - A_{1} + A_{2} {\rm X}^{2} }}{{6{\rm X}^{4} }}\cos 2{\rm X}\,t + \frac{{ - A_{3} + A_{4} \sigma^{2} }}{{32{\rm X}^{5} }}\sin 3{\rm X}\,t$$

Once more, by replacing Eqs. (74) and (79), one discovers that the secular elements must be omitted in Eq. (76).

80 $$\varpi_{2} = \frac{{63A_{3}^{2} - {\rm X}^{2} (150A_{3} A_{4} + 320A_{1}^{2} ) + {\rm X}^{4} (352A_{1} A_{2} + 87A_{4}^{2} ) - 32A_{2}^{2} {\rm X}^{6} }}{{384{\rm X}^{6} }}.$$

The uniform formula of $\Lambda_{2} (t)$ can be designated as:

81 $$\Lambda_{2} (t) = \frac{ - 1}{{46080\sigma^{9} }}\left( \begin{gathered} \tilde{A}_{1} + \tilde{A}_{2} \sin {\rm X}\,t + \tilde{A}_{3} \cos {\rm X}\,t + \tilde{A}_{4} \sin 2{\rm X}\,t + \tilde{A}_{5} \cos 2{\rm X}\,t + \hfill \\ \tilde{A}_{6} \sin 3{\rm X}\,t + \tilde{A}_{7} \cos 3{\rm X}\,t + \tilde{A}_{8} \cos 4{\rm X}\,t + \tilde{A}_{9} \sin 5{\rm X}\,t \hfill \\ \end{gathered} \right).$$

Substituting Eqs. (74), (79) and (81) into Eq. (77), the cancelation of the secular formulas requires

82 $$\varpi_{3} = \frac{1}{{512{\rm X}^{10} }}\left( \begin{gathered} 33A_{3}^{3} + {\rm X}^{2} (240A_{1}^{2} A_{3} - 150A_{3}^{2} A_{4} ) + {\rm X}^{4} ( - 392A_{1} A_{2} A_{3} + 336A_{1}^{2} A_{4} + 217A_{4}^{2} A_{3} ) \hfill \\ + {\rm X}^{6} ( - 408A_{1} A_{2} A_{4} + 184A_{2}^{2} A_{3} - 100A_{4}^{3} ) + 40A_{2}^{2} A_{4} {\rm X}^{8} \hfill \\ \end{gathered} \right).$$

The solution of $\Lambda_{3} (t)$ can be designated as:

83 $$\Lambda_{3} (t) = \frac{ - 1}{{{ 154828800}{\rm X}^{13} }}\left( \begin{gathered} \tilde{A}_{10} + \tilde{A}_{11} \sin {\rm X}\,t + \tilde{A}_{12} \cos {\rm X}\,t + \tilde{A}_{13} \sin 2{\rm X}\,t + \tilde{A}_{14} \cos 2{\rm X}\,t + \tilde{A}_{15} \sin 3{\rm X}\,t \hfill \\ + \tilde{A}_{16} \cos 3{\rm X}\,t + \tilde{A}_{17} \sin 4{\rm X}\,t + \tilde{A}_{18} \cos 4{\rm X}\,t + \tilde{A}_{19} \sin 5{\rm X}\,t + \tilde{A}_{20} \cos 5{\rm X}\,t \hfill \\ + \tilde{A}_{21} \cos 6{\rm X}\,t \hfill \\ \end{gathered} \right)$$

where $\tilde{A}_{i} ,\,(i = 1,2,....)$ is obvious from the context, so we won't include it to save time.

Last but not least, Eq. (70) is provided as:

84 $$\Lambda (t) = \mathop {lim}\limits_{\delta \to 1} \left( {\Lambda_{0} (t) + \delta \Lambda_{1} (t) + \delta^{2} \Lambda_{2} (t) + \delta^{3} \Lambda_{3} (t)} \right),$$

where $\Lambda_{0} (t)\,,\,\Lambda_{1} (t)$ and $\Lambda_{2} (t)$ are given by Eqs. (79), (81) and (83), correspondingly.

At this stage, replacing Eqs. (78), (80) and (82) in Eq. (69), an equation of sixth degree in ${\rm X}^{2}$ is given as:

85 $${\rm X}^{12} + \tilde{s}_{1} {\rm X}^{10} + \tilde{s}_{2} {\rm X}^{8} + \tilde{s}_{3} {\rm X}^{6} + \tilde{s}_{4} {\rm X}^{4} + \tilde{s}_{5} {\rm X}^{2} - \tilde{s}_{6} = 0,$$

where $\tilde{s}_{i}$ are factors which are signified from the framework.

To confirm the previous approximate analytical solution, this solution will be matched with the numerical solution based on the RK-4. For this objective, the characteristic equation of the nonlinear expanded frequency will be solved by means of the Mathematica software. Therefore, the values of ${\rm X}$ are computed mathematically and have twelve roots. This equation has been computed mathematically to have twelve roots, of which the real positive one is selected. For appropriateness, a numerical estimation of $\eta (z,t) = \Lambda (t)e^{ikz}$ is graphed. To adopt the former formulation coming out in Eq. (84), we are obliged to provide a further example scheme. To confirm the inequality, we suppose that:

$$\begin{gathered} a = 0.2,\,b = 1.3,\,\rho_{1} = 0.1,\,\rho_{2} = 1.2,\,\alpha_{1} = 0.2,,\,V_{1} = 0.2,\,V_{2} = 0.3,\,\,\beta_{1} = 0.8,\,\,\beta_{2} = 0.9,\,\,\varepsilon_{2} = 0.5,\,\,\varepsilon_{1} = 0.2, \hfill \\ \,\,\,\,\,\,\,\,\,\,\,\,\,\,\,\,\,\,\,\,\,\,\,\,\,\,\,\,\,\,\,\,\,\,\,\,\gamma_{1} = 0.2,\,\,\gamma_{2} = 0.3,\,\xi_{2} = 0.8,\,\xi_{1} = 0.6,\,\,k = 0.1,\,\,m = 0.2,\,\,E^{2} = 60. \hfill \\ \end{gathered}$$

**Figure 13:**
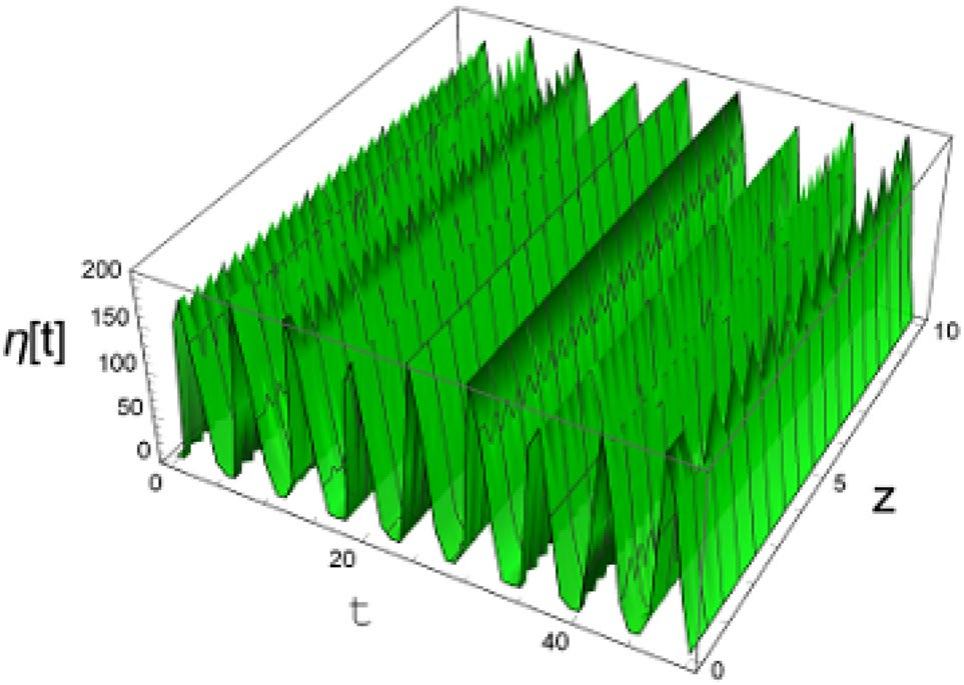
The estimate uniform solution of the surface deflection as given in Eq. (84).

**Figure 14:**
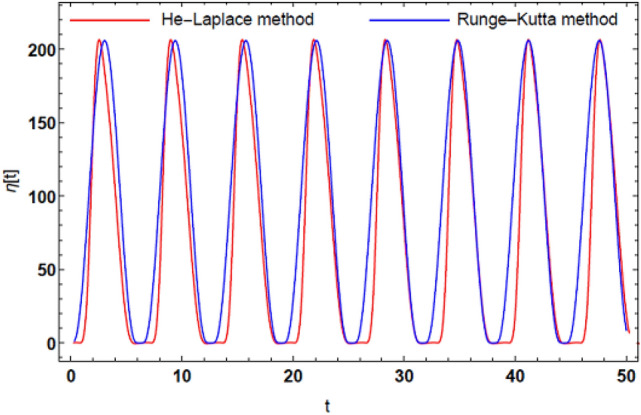
comparison between He-Laplace method and RK-4.

Thence, Fig. 13 illustrates chart $\Lambda (t)$ as shown in Eq. (84). This figure is delineated for $\eta (z,t) = \Lambda (t)e^{ikx}$ in three dimensions. Time and coordinate $z$ stand for the horizontal axes, where $z \in \left[ {0,\,\,10} \right]$. The surface displacement is graphed where $\eta \in \left[ {0,\,\,200} \right]$. It is indicated that $\eta (z,t)$ has a periodic performance in the $3\,D$ diagram. The matching between the approximate and numerical solution is presented in Fig. 14. This figure shows a good uniformity between the two solutions.

## Conclusions

The current manuscript scrutinizes the linear/nonlinear stability of a cylinder-shaped interface that divides two homogenous, incompressible, viscous flowing liquids. A constant axial electric field affects the system. Additionally considered are the media porosities that are implemented. The result of MHT is complicated in this investigation in line with the widespread applications of immiscible liquids. On the contrary, because of the influence of the linear theory, control factors of this transfer have been brought about by the nonlinear analysis. The analysis of the issue has followed the streamlined format of Hsieh^11,12^ for easier comprehension. A parameter of this transmission has been produced using the linear manner in light of this effect. However, the nonlinear analysis has produced two transmission factors. A partial differential equation in a nonlinear form of the surface displacement has been produced by the study. When the nonlinear elements are ignored, the linear stability requirements have been developed as a specific case. A series of graphics has been used to graphically represent these conditions. Some physical effects have been already noted. The nonlinear stability criteria have been also determined by driving the nonlinear Ginzburg–Landau equation. Through a series of figures, the actions of the different material elements on the stability of the interface have been addressed. To produce a bounded solution to a second-order differential equation, the idea of the nonlinear extended frequency as well as the coupling of the Laplace transforms and HPM have been further addressed. This equation indicates a complex, highly nonlinear equation. The Helmholtz-Duffing Eq. (66) is given by combining the real and imaginary components since the surface wave amplitude exhibits real behavior. The surface displacement profile has been examined, and the RK-4 has been numerically compared. The following conclusions can be derived from the results:

- Linear stability analysis

1. It is indicated that Weber’s number and the MHT coefficient have a destabilizing impact.
2. The Ohnesorge number as well as the Darcy numeral have destabilizing influences on the considered system.

- Nonlinear stability investigation

1. The computations demonstrated that the transition curves, which are specified by Eqs. (62) and (63), detached the zone of stability into two parts. Actually, the new zone of instability is granted by the nonlinear mechanism. So, the stable zone lies only between the two curves, otherwise the system becomes unstable.
2. The modulation of the nonlinearity exhibits some factors such as cross viscosity of Reiner–Rivlin type $\beta$ besides the MHT formulas $\alpha_{2}$ and $\alpha_{3}$ in the stability profile.
3. The nonlinear formulas of heat and mass transfer $\alpha_{2}$ and $\alpha_{3}$ as well as the cross viscosity exhibit a stabilizing impact.
4. The Darcy factor and the linear proportion viscosity have destabilizing influences.

- The surface deflection profile

It is indicated that $\eta (z,t)$ has a regular performance in the $3\,D$ drawing. Additionally, the two-dimensional solution matches with the numerical one.

As recommendations for further studies, the following portions will be mentioned in subsequent papers:

1. The presence of the energy and concentration equations will be analyzed away from the Hsieh's simplified formulation.
2. A variety of non-Newtonian fluid types, including nanofluids, will be employed.
3. Distinct stability problems in plane geometry will be discussed in light of the significance of various practical engineering applications.
4. An external field that changes over time will be provided.
5. Via the non-perturbative methodologies employed in assessing the nonlinear PDEs, it is possible to investigate the stability technique using a non-perturbative improved system.

## Data Availability

All data generated or analyzed during this study are included in this manuscript.

## Appendix

The constants of Eq. (45) may be listed as follows:

$${g}_{1}={I}_{0}(kR){K}_{1}(ka)+{I}_{1}(ka){K}_{0}(kR),$$

$${g}_{2}={I}_{0}(kR){K}_{1}(kb)+{I}_{1}(kb){K}_{0}(kR),$$

$${f}_{1}={I}_{1}(kR){K}_{1}(ka)-{I}_{1}(ka){K}_{1}(kR),$$

$${f}_{2}={I}_{1}(kR){K}_{1}(kb)-{I}_{1}(kb){K}_{1}(kR),$$

$${a}_{1}={g}_{2}{f}_{1}{\epsilon }_{1}+{g}_{1}{f}_{2}{\epsilon }_{2},$$

$${a}_{2}=\left({f}_{1}{f}_{2}+{g}_{1}{g}_{2}\right)\left({\epsilon }_{2}-{\epsilon }_{1}\right),$$

$${a}_{3}={g}_{2}{f}_{1}{\epsilon }_{2}-{g}_{1}{f}_{2}{\epsilon }_{1},$$

$${c}_{1}=\frac{{g}_{1}{\rho }_{1}}{k{f}_{1}}-\frac{{g}_{2}{\rho }_{2}}{k{f}_{2}},$$

$${c}_{2}=\frac{{\alpha }_{1}}{k}\left(\frac{{g}_{1}}{{f}_{1}}-\frac{{g}_{2}}{{f}_{2}}\right)-\frac{2}{R}\left({\gamma }_{1}{m}_{1}-{\gamma }_{2}{m}_{2}\right)+2k\left(\frac{{g}_{1}{m}_{1}{\gamma }_{1}}{{f}_{1}}-\frac{{g}_{2}{m}_{2}{\gamma }_{2}}{{f}_{2}}\right)+\frac{1}{k}\left(\frac{{g}_{1}{m}_{1}{\xi }_{1}}{{f}_{1}}-\frac{{g}_{2}{m}_{2}{\xi }_{2}}{{f}_{2}}\right),$$

$${d}_{1}=\left(\frac{{g}_{1}{v}_{1}{\rho }_{1}}{{f}_{1}{m}_{1}}-\frac{{g}_{2}{v}_{2}{\rho }_{2}}{{f}_{2}{m}_{2}}\right),$$

$${c}_{3}=\frac{{g}_{1}{v}_{1}{\rho }_{1}}{k{f}_{1}{m}_{1}}-\frac{{g}_{2}{v}_{2}{\rho }_{2}}{k{f}_{2}{m}_{2}},$$

$${c}_{4}=-\frac{\sigma }{{R}^{2}}-2\frac{{\alpha }_{1}}{R}\left(\frac{{m}_{1}{\gamma }_{1}}{{\rho }_{1}}-\frac{{m}_{2}{\gamma }_{2}}{{\rho }_{2}}\right)+2k{\alpha }_{1}\left(\frac{{g}_{1}{m}_{1}{\gamma }_{1}}{{f}_{1}{\rho }_{1}}-\frac{{g}_{2}{m}_{2}{\gamma }_{2}}{{f}_{2}{\rho }_{2}}\right)+\frac{{\alpha }_{1}}{k}\left(\frac{{g}_{1}{m}_{1}{\xi }_{1}}{{f}_{1}{\rho }_{1}}-\frac{{g}_{2}{m}_{2}{\xi }_{2}}{{f}_{2}{\rho }_{2}}\right)$$

$${d}_{2}={\alpha }_{1}\left(\frac{{g}_{1}{v}_{1}}{{f}_{1}{m}_{1}}-\frac{{g}_{2}{v}_{2}}{{f}_{2}{m}_{2}}\right),$$

$${c}_{5}=-\frac{2}{R}\left({\gamma }_{1}{v}_{1}-{\gamma }_{2}{v}_{2}\right)+2k\left(\frac{{g}_{1}{v}_{1}{\gamma }_{1}}{{f}_{1}}-\frac{{g}_{2}{v}_{2}{\gamma }_{2}}{{f}_{2}}\right)+\frac{1}{k}\left(\frac{{g}_{1}{v}_{1}{\xi }_{1}}{{f}_{1}}-\frac{{g}_{2}{v}_{2}{\xi }_{2}}{{f}_{2}}\right),$$

$${d}_{31}=\left(\frac{{g}_{1}{v}_{1}^{2}{\rho }_{1}}{{f}_{1}{m}_{1}^{2}}-\frac{{g}_{2}{v}_{2}^{2}{\rho }_{2}}{{f}_{2}{m}_{2}^{2}}\right),$$

$${d}_{32}=-\frac{1}{{a}_{1}}{g}_{1}{g}_{2}{\left({\epsilon }_{1}-{\epsilon }_{2}\right)}^{2},$$

$${c}_{6}=4\left({\beta }_{1}{m}_{1}^{2}-{\beta }_{2}{m}_{2}^{2}\right)-\frac{8k}{R}\left(\frac{{g}_{1}{m}_{1}^{2}{\beta }_{1}}{{f}_{1}}-\frac{{g}_{2}{m}_{2}^{2}{\beta }_{2}}{{f}_{2}}\right)+4{k}^{2}\left(\frac{{g}_{1}^{2}{m}_{1}^{2}{\beta }_{1}}{{f}_{1}^{2}}-\frac{{g}_{2}^{2}{m}_{2}^{2}{\beta }_{2}}{{f}_{2}^{2}}\right),$$

$${c}_{7}=\frac{\sigma }{{R}^{3}}+4{\alpha }_{1}^{2}(\left(-{k}^{2}+\frac{1}{{R}^{2}}\right)\left(\frac{{\beta }_{1}{m}_{1}^{2}}{{\rho }_{1}^{2}}-\frac{{\beta }_{2}{m}_{2}^{2}}{{\rho }_{2}^{2}}\right)-\frac{2k}{R}\left(\frac{{\beta }_{1}{g}_{1}{m}_{1}^{2}}{{f}_{1}{\rho }_{1}^{2}}-\frac{{\beta }_{2}{g}_{2}{m}_{2}^{2}}{{f}_{2}{\rho }_{2}^{2}}\right)+{k}^{2}\left(\frac{{\beta }_{1}{g}_{1}^{2}{m}_{1}^{2}}{{f}_{1}^{2}{\rho }_{1}^{2}}-\frac{{\beta }_{2}{g}_{2}^{2}{m}_{2}^{2}}{{f}_{2}^{2}{\rho }_{2}^{2}}\right))-\frac{{\alpha }_{1}^{2}}{k}\left(\frac{{g}_{1}}{{f}_{1}{\rho }_{1}}-\frac{{g}_{2}}{k{f}_{2}{\rho }_{2}}\right)-\frac{2{\alpha }_{1}{\alpha }_{2}}{R}\left(\frac{{\gamma }_{1}{m}_{1}}{{\rho }_{1}}-\frac{{\gamma }_{2}{m}_{2}}{{\rho }_{2}}\right)+2k{\alpha }_{1}{\alpha }_{2}+\frac{{\alpha }_{1}{\alpha }_{2}}{k}\left(\frac{{g}_{1}{m}_{1}{\xi }_{1}}{{f}_{1}{\rho }_{1}}-\frac{{g}_{2}{m}_{2}{\xi }_{2}}{{f}_{2}{\rho }_{2}}\right),$$

$${d}_{4}={\alpha }_{1}{\alpha }_{2}\left(\frac{{g}_{1}{v}_{1}}{{f}_{1}{m}_{1}}-\frac{{g}_{2}{v}_{2}}{{f}_{2}{m}_{2}}\right),$$

$${c}_{81}=\frac{\sigma }{2R}-{\Gamma }_{1}+{\Gamma }_{2}+\left({\rho }_{1}-{\rho }_{2}\right)+\left({v}_{1}{\xi }_{1}-{v}_{2}{\xi }_{2}\right)-\frac{{g}_{1}^{2}{v}_{1}^{2}{\rho }_{1}}{{f}_{1}^{2}{m}_{1}^{2}}+\frac{{g}_{2}^{2}{v}_{2}^{2}{\rho }_{2}}{{f}_{2}^{2}{m}_{2}^{2}}-4\left(-{k}^{2}+\frac{1}{{R}^{2}}\right)\left({\beta }_{1}{v}_{1}^{2}-{\beta }_{2}{v}_{2}^{2}\right)-\frac{8k}{R}\left(\frac{{\beta }_{1}{g}_{1}{v}_{1}^{2}}{{f}_{1}}-\frac{{\beta }_{2}{g}_{2}{v}_{2}^{2}}{{f}_{2}}\right)+4{k}^{2}\left(\frac{{\beta }_{1}{g}_{1}^{2}{v}_{1}^{2}}{{f}_{1}^{2}}-\frac{{\beta }_{2}{g}_{2}^{2}{v}_{2}^{2}}{{f}_{2}^{2}}\right),$$

$${c}_{82}=(\frac{1}{2}\left({\epsilon }_{1}-{\epsilon }_{2}\right)-\frac{{a}_{2}{g}_{1}{g}_{2}{\left({\epsilon }_{1}-{\epsilon }_{2}\right)}^{2}}{{a}_{1}^{2}}+\frac{{g}_{1}^{2}{g}_{2}^{2}{\left({\epsilon }_{1}-{\epsilon }_{2}\right)}^{3}}{2{a}_{1}^{2}}+\frac{1}{2{k}^{2}{a}_{1}^{2}}({f}_{1}^{2}{g}_{2}^{2}{\epsilon }_{1}^{3}-{f}_{2}^{2}{g}_{1}^{2}{\epsilon }_{2}^{3}-\left(-{f}_{2}^{2}{g}_{1}^{2}-2{f}_{1}^{2}{g}_{2}^{2}\right){\epsilon }_{1}^{2}{\epsilon }_{2}+({f}_{1}^{2}{g}_{2}^{2}+2{f}_{2}^{2}{g}_{1}^{2}){\epsilon }_{1}{\epsilon }_{2}^{2})-\frac{1}{{a}_{1}}({f}_{2}{g}_{1}{\epsilon }_{1}^{2}+{f}_{1}{g}_{2}{\epsilon }_{2}^{2}-\left({f}_{2}{g}_{1}+{f}_{1}{g}_{2}\right){\epsilon }_{1}{\epsilon }_{2}+\frac{2}{k}\left({f}_{1}{g}_{2}{\epsilon }_{1}^{2}+{f}_{2}{g}_{1}{\epsilon }_{2}^{2}-\left({f}_{2}{g}_{1}+{f}_{1}{g}_{2}\right){\epsilon }_{1}{\epsilon }_{2}\right))),$$

$${d}_{5}=\left(\frac{1}{k}\left(\frac{{g}_{1}^{2}{v}_{1}{\xi }_{1}}{{f}_{1}^{2}}-\frac{{g}_{2}^{2}{v}_{2}{\xi }_{2}}{{f}_{2}^{2}}\right)-4k\left({\gamma }_{1}{v}_{1}-{\gamma }_{2}{v}_{2}\right)-\frac{2}{R}\left(\frac{{\gamma }_{1}{g}_{1}{v}_{1}}{{f}_{1}}-\frac{{\gamma }_{2}{g}_{2}{v}_{2}}{{f}_{2}}\right)+2k\left(\frac{{g}_{1}^{2}{v}_{1}{\gamma }_{1}}{{f}_{1}^{2}}-\frac{{g}_{2}^{2}{v}_{2}{\gamma }_{2}}{{f}_{2}^{2}}\right)\right),$$

$${c}_{9}=-\frac{{g}_{1}^{2}{v}_{1}{\alpha }_{1}}{{f}_{1}^{2}{m}_{1}}+\frac{{g}_{2}^{2}{v}_{2}{\alpha }_{1}}{{f}_{2}^{2}{m}_{2}}+\left(\frac{{v}_{1}}{{m}_{1}}-\frac{{v}_{2}}{{m}_{2}}-\frac{1}{k}\left(\frac{{g}_{1}{v}_{1}}{{f}_{1}{m}_{1}}-\frac{{g}_{2}{v}_{2}}{{f}_{2}{m}_{2}}\right)\right){\alpha }_{1}+8{\alpha }_{1}{k}^{2}\left(-\frac{{\beta }_{1}{m}_{1}{v}_{1}}{{\rho }_{1}}+\frac{{\beta }_{2}{m}_{2}{v}_{2}}{{\rho }_{2}}+\frac{{\beta }_{1}{\left(-\frac{{f}_{1}}{kR}+{g}_{1}\right)}^{2}{m}_{1}{v}_{1}}{{f}_{1}^{2}{\rho }_{1}}-\frac{{\beta }_{2}{\left(-\frac{{f}_{2}}{kR}+{g}_{2}\right)}^{2}{m}_{2}{v}_{2}}{{f}_{2}^{2}{\rho }_{2}}\right),$$

$${d}_{6}=2k{\alpha }_{1}\left(\frac{{g}_{1}{\gamma }_{1}\left(-\frac{{f}_{1}}{kR}+{g}_{1}\right){m}_{1}}{{f}_{1}^{2}{\rho }_{1}}-\frac{{g}_{2}{\gamma }_{2}\left(-\frac{{f}_{2}}{kR}+{g}_{2}\right){m}_{2}}{{f}_{2}^{2}{\rho }_{2}}\right)+\frac{{\alpha }_{1}}{k}\left(\frac{{g}_{1}^{2}{m}_{1}{\xi }_{1}}{{f}_{1}^{2}{\rho }_{1}}-\frac{{g}_{2}^{2}{m}_{2}{\xi }_{2}}{{f}_{2}^{2}{\rho }_{2}}\right)-4k{\alpha }_{1}\left(\frac{{\gamma }_{1}{m}_{1}}{{\rho }_{1}}-\frac{{\gamma }_{2}{m}_{2}}{{\rho }_{2}}\right),$$

$${c}_{10}=-8{k}^{2}\left({\beta }_{1}{m}_{1}{v}_{1}-{\beta }_{2}{m}_{2}{v}_{2}-\frac{{\beta }_{1}{\left(-\frac{{f}_{1}}{kR}+{g}_{1}\right)}^{2}{m}_{1}{v}_{1}}{{f}_{1}^{2}}-\frac{{\beta }_{2}{\left(-\frac{{f}_{2}}{kR}+{g}_{2}\right)}^{2}{m}_{2}{v}_{2}}{{f}_{2}^{2}}\right)-\left(\frac{{g}_{1}^{2}{v}_{1}}{{m}_{1}{f}_{1}^{2}}-\frac{{g}_{2}^{2}{v}_{2}}{{m}_{2}{f}_{2}^{2}}\right),$$

$${d}_{7}=\frac{1}{k}\left(\frac{{g}_{1}^{2}{m}_{1}{\xi }_{1}}{{f}_{1}^{2}}-\frac{{g}_{2}^{2}{m}_{2}{\xi }_{2}}{{f}_{2}^{2}}+\frac{{g}_{1}^{2}{\alpha }_{1}}{{f}_{1}^{2}}-\frac{{g}_{2}^{2}{\alpha }_{1}}{{f}_{2}^{2}}\right)+2k\left(\frac{{\gamma }_{1}{g}_{1}\left(-\frac{{f}_{1}}{kR}+{g}_{1}\right){m}_{1}}{{f}_{1}^{2}}-\frac{{\gamma }_{2}{g}_{2}\left(-\frac{{f}_{2}}{kR}+{g}_{2}\right){m}_{2}}{{f}_{2}^{2}}\right)-4k\left({\gamma }_{1}{m}_{1}-{\gamma }_{2}{m}_{2}\right),$$

$${c}_{11}=\frac{{\alpha }_{1}}{k}\left(-\frac{{g}_{1}}{{f}_{1}}+\frac{{g}_{2}}{{f}_{2}}\right)-8{\alpha }_{1}(\frac{{\beta }_{1}{m}_{1}^{2}}{{\rho }_{1}}-\frac{{\beta }_{2}{m}_{2}^{2}}{{\rho }_{2}}-\frac{{\beta }_{1}{\left(-\frac{{f}_{1}}{kR}+{g}_{1}\right)}^{2}{m}_{1}^{2}}{{f}_{1}^{2}{\rho }_{1}}+\frac{{\beta }_{2}{\left(-\frac{{f}_{2}}{kR}+{g}_{2}\right)}^{2}{m}_{2}^{2}}{{f}_{2}^{2}{\rho }_{2}}),$$

$${d}_{8}=\frac{1}{k}\left(\frac{{g}_{1}^{2}{v}_{1}{\rho }_{1}}{{f}_{1}^{2}{m}_{1}}-\frac{{g}_{2}^{2}{v}_{2}{\rho }_{2}}{{f}_{2}^{2}{m}_{2}}\right),$$

$${d}_{9}=\frac{1}{k}\left(\frac{{g}_{1}^{2}{\rho }_{1}}{{f}_{1}^{2}}-\frac{{g}_{2}^{2}{\rho }_{2}}{{f}_{2}^{2}}\right),$$

$${c}_{12}=\frac{1}{k}\left(-\frac{{g}_{1}^{3}{\rho }_{1}}{{f}_{1}^{3}}+\frac{{g}_{2}^{3}{\rho }_{2}}{{f}_{2}^{3}}+\frac{{g}_{1}{\rho }_{1}}{{f}_{1}}-\frac{{g}_{2}{\rho }_{2}}{{f}_{2}}\right),$$

$${c}_{13}=\frac{1}{k}\left(-\frac{{g}_{1}}{{f}_{1}}+\frac{{g}_{2}}{{f}_{2}}\right){\alpha }_{1}{\alpha }_{2}+8{k}^{2}{\alpha }_{1}{\alpha }_{2}(-\frac{{\beta }_{1}{m}_{1}^{2}}{{\rho }_{1}}+\frac{{\beta }_{2}{m}_{2}^{2}}{{\rho }_{2}}+\frac{{\beta }_{1}{m}_{1}^{2}{\left(-\frac{{f}_{1}}{kR}+{g}_{1}\right)}^{2}}{{f}_{1}^{2}{\rho }_{1}}+\frac{{\beta }_{2}{m}_{2}^{2}{\left(-\frac{{f}_{2}}{kR}+{g}_{2}\right)}^{2}}{{f}_{2}^{2}{\rho }_{2}}),$$

$${c}_{14}=\frac{1}{k}\left(-\frac{{g}_{1}^{3}{v}_{1}{\rho }_{1}}{{f}_{1}^{3}{m}_{1}}+\frac{{g}_{2}^{3}{v}_{2}{\rho }_{2}}{{f}_{2}^{3}{m}_{2}}+\frac{{g}_{1}{v}_{1}{\rho }_{1}}{{f}_{1}{m}_{1}}-\frac{{g}_{2}{v}_{2}{\rho }_{2}}{{f}_{2}{m}_{2}}\right),$$

$${c}_{15}=-2k\left(\frac{{\gamma }_{1}{g}_{1}^{2}{m}_{1}\left(-\frac{{f}_{1}}{kR}+{g}_{1}\right)}{{f}_{1}^{3}}-\frac{{\gamma }_{2}{g}_{2}^{2}{m}_{2}\left(-\frac{{f}_{2}}{kR}+{g}_{2}\right)}{{f}_{2}^{3}}-2\left(\frac{{\gamma }_{1}{g}_{1}{m}_{1}}{{f}_{1}}+\frac{{\gamma }_{2}{g}_{2}{m}_{2}}{{f}_{2}}\right)\right)-\frac{1}{k}\left(\frac{{g}_{1}^{3}{m}_{1}{\xi }_{1}}{{f}_{1}^{3}}+\frac{{g}_{2}^{3}{m}_{2}{\xi }_{2}}{{f}_{2}^{3}}\right)-\frac{{\alpha }_{1}}{k}\left(\frac{{g}_{1}^{3}}{{f}_{1}^{3}}-\frac{{g}_{2}^{3}}{{f}_{2}^{3}}\right)-2k\left(\frac{{\gamma }_{1}{g}_{1}{m}_{1}}{{f}_{1}}-\frac{{\gamma }_{2}{g}_{2}{m}_{2}}{{f}_{2}}\right)+\frac{1}{k}\left(\frac{{g}_{1}{m}_{1}{\xi }_{1}}{{f}_{1}}-\frac{{g}_{2}{m}_{2}{\xi }_{2}}{{f}_{2}}\right)+\frac{{\alpha }_{1}}{k}\left(\frac{{g}_{1}}{{f}_{1}}-\frac{{g}_{2}}{{f}_{2}}\right),$$

$${d}_{11}={\alpha }_{1}\left(\frac{{g}_{1}}{{f}_{1}}-\frac{{g}_{2}}{{f}_{2}}-\frac{1}{k}\left(\frac{{g}_{1}^{2}}{{f}_{1}^{2}}-\frac{{g}_{2}^{2}}{{f}_{2}^{2}}\right)\right)+16{k}^{2}{\alpha }_{1}(-\frac{{\beta }_{1}{g}_{1}{m}_{1}^{2}}{{f}_{1}{\rho }_{1}}+\frac{{\beta }_{2}{g}_{2}{m}_{2}^{2}}{{f}_{2}{\rho }_{2}}-\frac{{\beta }_{2}{g}_{2}{\left(-\frac{{f}_{2}}{kR}+{g}_{2}\right)}^{2}{m}_{2}^{2}}{{f}_{2}^{3}{\rho }_{2}}+\frac{{\beta }_{1}{g}_{1}{\left(-\frac{{f}_{1}}{kR}+{g}_{1}\right)}^{2}{m}_{1}^{2}}{{f}_{1}^{3}{\rho }_{1}})$$

$${d}_{12}=8{k}^{2}(-\frac{{\beta }_{1}{g}_{1}{m}_{1}^{2}}{{f}_{1}}+\frac{{\beta }_{2}{g}_{2}{m}_{2}^{2}}{{f}_{2}}-\frac{{\beta }_{2}{g}_{2}{m}_{2}^{2}{\left(-\frac{{f}_{2}}{kR}+{g}_{2}\right)}^{2}}{{f}_{2}^{3}}+\frac{{\beta }_{1}{g}_{1}{m}_{1}^{2}{\left(-\frac{{f}_{1}}{kR}+{g}_{1}\right)}^{2}}{{f}_{1}^{3}})$$

$${c}_{16}=-\frac{\sigma }{{R}^{4}}-8{\alpha }_{1}^{2}{\alpha }_{2}\left({k}^{2}-\frac{1}{{R}^{2}}\right)\left(\frac{{\beta }_{1}{m}_{1}^{2}}{{\rho }_{1}^{2}}-\frac{{\beta }_{1}{m}_{2}^{2}}{{\rho }_{2}^{2}}\right)-\frac{16k{\alpha }_{1}^{2}{\alpha }_{2}}{R}\left(\frac{{\beta }_{1}{g}_{1}{m}_{1}^{2}}{{f}_{1}{\rho }_{1}^{2}}-\frac{{\beta }_{2}{g}_{2}{m}_{2}^{2}}{{f}_{2}{\rho }_{2}^{2}}\right)+8{k}^{2}{\alpha }_{1}^{2}{\alpha }_{2}\left(\frac{{\beta }_{1}{g}_{1}^{2}{m}_{1}^{2}}{{f}_{1}^{2}{\rho }_{1}^{2}}-\frac{{\beta }_{1}{g}_{2}^{2}{m}_{2}^{2}}{{f}_{2}^{2}{\rho }_{2}^{2}}\right)-\frac{2{\alpha }_{1}^{2}{\alpha }_{2}}{k}\left(\frac{{g}_{1}}{{f}_{1}{\rho }_{1}}-\frac{{g}_{2}}{{f}_{2}{\rho }_{2}}\right)-\frac{2{\alpha }_{1}{\alpha }_{3}}{R}\left(\frac{{\gamma }_{1}{m}_{1}}{{\rho }_{1}}-\frac{{\gamma }_{2}{m}_{2}}{{\rho }_{2}}\right)+2k{\alpha }_{1}{\alpha }_{3}\left(\frac{{\gamma }_{1}{g}_{1}{m}_{1}}{{f}_{1}{\rho }_{1}}-\frac{{\gamma }_{2}{g}_{2}{m}_{2}}{{f}_{2}{\rho }_{2}}\right)+\frac{{\alpha }_{1}{\alpha }_{3}}{k}\left(\frac{{g}_{1}{m}_{1}{\xi }_{1}}{{f}_{1}{\rho }_{1}}-\frac{{g}_{2}{m}_{2}{\xi }_{2}}{{f}_{2}{\rho }_{2}}\right),$$

$${d}_{13}={\alpha }_{1}{\alpha }_{3}\left(\frac{{g}_{1}{v}_{1}}{{f}_{1}{m}_{1}}-\frac{{g}_{2}{v}_{2}}{{f}_{2}{m}_{2}}\right),$$

$${c}_{17}=2k\left(\frac{{\gamma }_{1}{g}_{1}{v}_{1}}{{f}_{1}}-\frac{{\gamma }_{2}{g}_{2}{v}_{2}}{{f}_{2}}-\frac{{\gamma }_{1}{g}_{1}^{3}{v}_{1}}{{f}_{1}^{3}}+\frac{{\gamma }_{2}{g}_{2}^{3}{v}_{2}}{{f}_{2}^{3}}\right)+\frac{2}{R}\left(\frac{{\gamma }_{1}{g}_{1}^{2}{v}_{1}}{{f}_{1}^{2}}-\frac{{\gamma }_{2}{g}_{2}^{2}{v}_{2}}{{f}_{2}^{2}}\right)+\frac{1}{k}\left(\frac{{g}_{1}{v}_{1}{\xi }_{1}}{{f}_{1}}-\frac{{g}_{2}{v}_{2}{\xi }_{2}}{{f}_{2}}-\frac{{g}_{1}^{3}{v}_{1}{\xi }_{1}}{{f}_{1}^{3}}+\frac{{g}_{2}^{3}{v}_{2}{\xi }_{2}}{{f}_{2}^{3}}\right),$$

$${d}_{141}=-8{k}^{2}\left(\frac{{\beta }_{1}{g}_{1}{v}_{1}^{2}}{{f}_{1}}-\frac{{\beta }_{2}{g}_{2}{v}_{2}^{2}}{{f}_{2}}-\frac{8{\beta }_{1}{g}_{1}^{3}{v}_{1}^{2}}{{f}_{1}^{3}}+\frac{{\beta }_{2}{g}_{2}^{3}{v}_{2}^{2}}{{f}_{2}^{3}}\right)+\frac{8}{{R}^{2}}\left(\frac{{\beta }_{1}{g}_{1}{v}_{1}^{2}{\gamma }_{2}}{{f}_{1}}-\frac{{\beta }_{2}{g}_{2}{v}_{2}^{2}}{{f}_{2}}\right)-\frac{16k}{R}\left(\frac{{\beta }_{1}{g}_{1}^{2}{v}_{1}^{2}}{{f}_{1}^{2}}-\frac{{\beta }_{2}{g}_{2}^{2}{v}_{2}^{2}}{{f}_{2}^{2}}\right)+\frac{{g}_{1}{v}_{1}^{2}{\rho }_{1}}{{f}_{1}{m}_{1}^{2}}-\frac{{g}_{2}{v}_{2}^{2}{\rho }_{2}}{{f}_{2}{m}_{2}^{2}}-\frac{{g}_{1}^{3}{v}_{1}^{2}{\rho }_{1}}{{f}_{1}^{3}{m}_{1}^{2}}+\frac{{g}_{2}^{3}{v}_{2}^{2}{\rho }_{2}}{{f}_{2}^{3}{m}_{2}^{2}}$$

$${d}_{142}=-\frac{1}{{k}^{2}{a}_{1}^{3}}\left({\epsilon }_{1}-{\epsilon }_{2}\right)(-k{a}_{1}^{2}\left(2{f}_{1}{f}_{2}+k{g}_{1}{g}_{2}\right)\left({\epsilon }_{1}-{\epsilon }_{2}\right)-{a}_{2}\left({\epsilon }_{1}-{\epsilon }_{2}\right)({k}^{2}{a}_{2}{g}_{1}{g}_{2}-\left({f}_{1}^{2}+{k}^{2}{g}_{1}^{2}\right){g}_{2}^{2}{\epsilon }_{1}+{g}_{1}^{2}\left({f}_{2}^{2}+{k}^{2}{g}_{2}^{2}\right){\epsilon }_{2})+{a}_{1}(\left({\epsilon }_{1}-{\epsilon }_{2}\right)({g}_{2}({f}_{1}^{2}{f}_{2}{\epsilon }_{1}+k{g}_{1}\left(-k{a}_{3}+\left(k{f}_{2}{g}_{1}+2{f}_{1}{g}_{2}\right){\epsilon }_{1}\right))-{g}_{1}\left(2k{f}_{2}{g}_{1}{g}_{2}+{f}_{1}\left({f}_{2}^{2}+{k}^{2}{g}_{2}^{2}\right)\right){\epsilon }_{2})+k{a}_{2}\left({f}_{2}{g}_{1}\left(-k{\epsilon }_{1}+2{\epsilon }_{2}\right)+{f}_{1}{g}_{2}\left(-2{\epsilon }_{1}+k{\epsilon }_{2}\right)\right)))$$

$${c}_{18}=-\frac{\sigma }{2{R}^{2}}-2k{\alpha }_{1}\left(\frac{{\gamma }_{1}{g}_{1}^{2}\left(-\frac{{f}_{1}}{kR}+{g}_{1}\right){m}_{1}}{{f}_{1}^{3}{\rho }_{1}}-\frac{{\gamma }_{2}{g}_{2}^{2}\left(-\frac{{f}_{2}}{kR}+{g}_{2}\right){m}_{2}}{{f}_{2}^{3}{\rho }_{2}}\right)-\frac{{\alpha }_{1}}{k}\left(\frac{{g}_{1}^{3}{m}_{1}{\xi }_{1}}{{f}_{1}^{3}{\rho }_{1}}+\frac{{g}_{2}^{3}{m}_{2}{\xi }_{2}}{{f}_{2}^{3}{\rho }_{2}}\right)+2k{\alpha }_{1}\left(\frac{{\gamma }_{1}{g}_{1}{m}_{1}}{{f}_{1}{\rho }_{1}}-\frac{{\gamma }_{2}{g}_{2}{m}_{2}}{{f}_{2}{\rho }_{2}}\right)-\frac{{\alpha }_{1}}{k}\left(\frac{{g}_{1}{m}_{1}{\xi }_{1}}{{f}_{1}{\rho }_{1}}+\frac{{g}_{2}{m}_{2}{\xi }_{2}}{{f}_{2}{\rho }_{2}}\right),$$

$${d}_{15}={\alpha }_{1}(-\left(\frac{{g}_{1}^{3}{v}_{1}}{{f}_{1}^{3}{m}_{1}}-\frac{{g}_{2}^{3}{v}_{2}}{{f}_{2}^{3}{m}_{2}}\right)-\frac{1}{k}\left(\frac{{g}_{1}^{2}{v}_{1}}{{f}_{1}^{2}{m}_{1}}-\frac{{g}_{2}^{2}{v}_{2}}{{f}_{2}^{2}{m}_{2}}\right)+\left(\frac{{g}_{1}{v}_{1}}{{f}_{1}{m}_{1}}-\frac{{g}_{2}{v}_{2}}{{f}_{2}{m}_{2}}\right)+16{k}^{2}(-\frac{{g}_{1}{\beta }_{1}{m}_{1}{v}_{1}}{{f}_{1}{\rho }_{1}}+\frac{{\beta }_{2}{g}_{2}{m}_{2}{v}_{2}}{{f}_{2}{\rho }_{2}}+\frac{{g}_{1}{\beta }_{1}{\left(-\frac{{f}_{1}}{kR}+{g}_{1}\right)}^{2}{m}_{1}{v}_{1}}{{f}_{1}^{3}{\rho }_{1}}-\frac{{g}_{2}{\beta }_{2}{\left(-\frac{{f}_{2}}{kR}+{g}_{2}\right)}^{2}{m}_{2}{v}_{2}}{{f}_{2}^{3}{\rho }_{2}})+\frac{{g}_{1}{v}_{1}}{{f}_{1}{m}_{1}}-\frac{{g}_{2}{v}_{2}}{{f}_{2}{m}_{2}}),$$

The constants of Eqs. (49) may be recorded as follows:

$${S}_{0}=-{c}_{1 }k,{S}_{1}=k\left({c}_{3}k+{d}_{1}\right),$$

$${b}_{1}=-{c}_{2}k,$$

$${S}_{21}=k\left({k}^{2}\sigma -{d}_{31}k+{c}_{4}\right),$$

$${S}_{22}=\frac{{k}^{2}}{{a}_{1}}{g}_{1}{g}_{2}{\left({\epsilon }_{1}-{\epsilon }_{2}\right)}^{2},$$

$${S}_{2}={S}_{21}+{E}_{0}^{2}{S}_{22},$$

$${b}_{2}=k\left({c}_{5}k+{d}_{2}\right),$$

Constants of Eq. (60):

$${s}_{3}=2\left(\frac{{g}_{2}{\rho }_{2}}{{f}_{2}}-\frac{{g}_{1}{\rho }_{1}}{{f}_{1}}\right)\omega -2k\left(\frac{{g}_{2}{\rho }_{2}{v}_{2}}{{m}_{2}{f}_{2}}-\frac{{g}_{1}{\rho }_{1}{v}_{1}}{{f}_{1}{m}_{1}}\right),$$

$${b}_{3}={\alpha }_{1}\left(\frac{{g}_{2}}{{f}_{2}}-\frac{{g}_{1}}{{f}_{1}}\right)+\frac{2k}{R}\left({m}_{1}{\gamma }_{1}-{m}_{2}{\gamma }_{2}\right)+2{k}^{2}\left(\frac{{g}_{2}{m}_{2}{\gamma }_{2}}{{f}_{2}}-\frac{{g}_{1}{m}_{1}{\gamma }_{1}}{{f}_{1}}\right)+\left(-\frac{{g}_{1}{m}_{1}{\xi }_{1}}{{f}_{1}}+\frac{{g}_{2}{m}_{2}{\xi }_{2}}{{f}_{2}}\right),$$

$${s}_{4}=\frac{{s}_{3}}{{{s}_{3}}^{2}+{{b}_{3}}^{2}},$$

$${s}_{5}=\frac{-{b}_{3}}{{{s}_{3}}^{2}+{{b}_{3}}^{2}},$$

$${s}_{6}=-2\left(\frac{{g}_{2}{\rho }_{2}{v}_{2}}{{m}_{2}{f}_{2}}-\frac{{g}_{1}{\rho }_{1}{v}_{1}}{{f}_{1}{m}_{1}}\right),$$

$${s}_{7}=\frac{2}{R}\left({m}_{1}{\gamma }_{1}-{m}_{2}{\gamma }_{2}\right)+4k\left(\frac{{g}_{2}{m}_{2}{\gamma }_{2}}{{f}_{2}}-\frac{{g}_{1}{m}_{1}{\gamma }_{1}}{{f}_{1}}\right),$$

$${s}_{8}=6k\sigma +2\left(\frac{{g}_{2}{\rho }_{2}{{v}_{2}}^{2}}{{{m}_{2}}^{2}{f}_{2}}-\frac{{g}_{1}{\rho }_{1}{{v}_{1}}^{2}}{{f}_{1}{{m}_{1}}^{2}}\right)-4{\alpha }_{1}\left(\frac{{g}_{2}{m}_{2}{\gamma }_{2}}{{f}_{2}{\rho }_{2}}-\frac{{g}_{1}{m}_{1}{\gamma }_{1}}{{f}_{1}{\rho }_{1}}\right),$$

$${s}_{9}=-12k\left(\frac{{\gamma }_{2}{g}_{2}{v}_{2}}{{f}_{2}}-\frac{{\gamma }_{1}{g}_{1}{v}_{1}}{{f}_{1}}\right)+4\left(\frac{{g}_{2}{m}_{2}{\gamma }_{2}}{{f}_{2}}-\frac{{g}_{1}{m}_{1}{\gamma }_{1}}{{f}_{1}}\right)-\frac{4}{R}\left({v}_{1}{\gamma }_{1}-{v}_{2}{\gamma }_{2}\right),$$

$${s}_{10}=\frac{2{g}_{1}{g}_{2}{\left({\epsilon }_{1}-{\epsilon }_{2}\right)}^{2}}{{g}_{2}{f}_{1}{\epsilon }_{1}-{g}_{1}{f}_{2}{\epsilon }_{2}},$$

$${s}_{11}=-2\left(\frac{{g}_{2}{\rho }_{2}{v}_{2}}{{m}_{2}{f}_{2}}-\frac{{g}_{1}{\rho }_{1}{v}_{1}}{{f}_{1}{m}_{1}}\right)\omega +3{k}^{2}\sigma -\frac{\sigma }{{R}^{2}}+\frac{2{\alpha }_{1}}{R}\left(\frac{{\gamma }_{2}{m}_{2}}{{\rho }_{2}}-\frac{{\gamma }_{1}{m}_{1}}{{\rho }_{1}}\right)+2k\left(\frac{{g}_{2}{\rho }_{2}{{v}_{2}}^{2}}{{{m}_{2}}^{2}{f}_{2}}-\frac{{g}_{1}{\rho }_{1}{{v}_{1}}^{2}}{{f}_{1}{{m}_{1}}^{2}}\right)-4k{\alpha }_{1}\left(\frac{{g}_{2}{m}_{2}{\gamma }_{2}}{{f}_{2}{\rho }_{2}}-\frac{{g}_{1}{m}_{1}{\gamma }_{1}}{{f}_{1}{\rho }_{1}}\right),$$

$${s}_{12}=\left(\frac{2}{R}\left({m}_{1}{\gamma }_{1}-{m}_{2}{\gamma }_{2}\right)-4k\left(\frac{{g}_{2}{m}_{2}{\gamma }_{2}}{{f}_{2}}-\frac{{g}_{1}{m}_{1}{\gamma }_{1}}{{f}_{1}}\right)\right)\omega -{\alpha }_{1}\left(\frac{{g}_{2}{v}_{2}}{{m}_{2}{f}_{2}}-\frac{{g}_{1}{v}_{1}}{{f}_{1}{m}_{1}}\right)-6{k}^{2}\left(\frac{{\gamma }_{2}{g}_{2}{v}_{2}}{{f}_{2}}-\frac{{\gamma }_{1}{g}_{1}{v}_{1}}{{f}_{1}}\right)+\left(\frac{{g}_{1}{v}_{1}{\xi }_{1}}{{f}_{1}}-\frac{{g}_{2}{v}_{2}{\xi }_{2}}{{f}_{2}}\right)-\frac{4k}{R}\left({v}_{1}{\gamma }_{1}-{v}_{2}{\gamma }_{2}\right),$$

$${s}_{13}=\frac{2{g}_{1}{g}_{2}k{\left({\epsilon }_{1}-{\epsilon }_{2}\right)}^{2}}{{g}_{2}{f}_{1}{\epsilon }_{1}-{g}_{1}{f}_{2}{\epsilon }_{2}},$$

$${h}_{1}=-{s}_{11}{s}_{4}+{s}_{12}{s}_{5},$$

$${h}_{2}=-{s}_{12}{s}_{4}-{s}_{11}{s}_{5},$$

$${h}_{3}=-{s}_{13}{s}_{4},$$

$${h}_{4}=-{s}_{13}{s}_{5},$$

$${D}_{3}=\frac{4{g}_{1}{g}_{2}{k}^{2}{\left({\epsilon }_{1}-{\epsilon }_{2}\right)}^{2}}{{g}_{2}{f}_{1}{\epsilon }_{1}-{g}_{1}{f}_{2}{\epsilon }_{2}},$$

$${D}_{1}=4\left(\frac{{g}_{2}{\rho }_{2}}{{f}_{2}}-\frac{{g}_{1}{\rho }_{1}}{{f}_{1}}\right){\omega }^{2}-8k\left(\frac{{g}_{2}{\rho }_{2}{v}_{2}}{{m}_{2}{f}_{2}}-\frac{{g}_{1}{\rho }_{1}{v}_{1}}{{f}_{1}{m}_{1}}\right)\omega +8{k}^{3}\sigma -\frac{2k\sigma }{{R}^{2}}+\frac{2k{\alpha }_{1}}{R}\left(\frac{{\gamma }_{2}{m}_{2}}{{\rho }_{2}}-\frac{{\gamma }_{1}{m}_{1}}{{\rho }_{1}}\right)+4{k}^{2}\left(\frac{{g}_{2}{\rho }_{2}{{v}_{2}}^{2}}{{{m}_{2}}^{2}{f}_{2}}-\frac{{g}_{1}{\rho }_{1}{{v}_{1}}^{2}}{{f}_{1}{{m}_{1}}^{2}}\right)-8{k}^{2}{\alpha }_{1}\left(\frac{{g}_{2}{m}_{2}{\gamma }_{2}}{{f}_{2}{\rho }_{2}}-\frac{{g}_{1}{m}_{1}{\gamma }_{1}}{{f}_{1}{\rho }_{1}}\right)+{\alpha }_{1}\left(\frac{{g}_{1}{m}_{1}{\xi }_{1}}{{f}_{1}{\rho }_{1}}-\frac{{g}_{2}{m}_{2}{\xi }_{2}}{{f}_{2}{\rho }_{2}}\right),$$

$${D}_{2}=2({\alpha }_{1}\left(\frac{{g}_{2}}{{f}_{2}}-\frac{{g}_{1}}{{f}_{1}}\right)+\frac{4k}{R}\left({m}_{1}{\gamma }_{1}-{m}_{2}{\gamma }_{2}\right)+8{k}^{2}\left(\frac{{g}_{2}{m}_{2}{\gamma }_{2}}{{f}_{2}}-\frac{{g}_{1}{m}_{1}{\gamma }_{1}}{{f}_{1}}\right)-\left(\frac{{g}_{1}{m}_{1}{\xi }_{1}}{{f}_{1}}-\frac{{g}_{2}{m}_{2}{\xi }_{2}}{{f}_{2}}\right))\omega -2k\left(\frac{{g}_{2}{v}_{2}{\gamma }_{2}}{{f}_{2}{m}_{2}}-\frac{{g}_{1}{v}_{1}{\gamma }_{1}}{{f}_{1}{m}_{1}}\right)+16{k}^{3}\left(-\frac{{g}_{2}{v}_{2}{\gamma }_{2}}{{f}_{2}}+\frac{{g}_{1}{v}_{1}{\gamma }_{1}}{{f}_{1}}\right)+\frac{8{k}^{2}}{R}\left({\gamma }_{2}{v}_{2}-{\gamma }_{1}{v}_{1}\right)+2k\left(\frac{{g}_{1}{v}_{1}{\xi }_{1}}{{f}_{1}}-\frac{{g}_{2}{v}_{2}{\xi }_{2}}{{f}_{2}}\right),$$

$${n}_{1}=k\left({d}_{9}k-{c}_{6}\right){\omega }^{2}-{k}^{2}\left({d}_{8}k-{c}_{10}\right)\omega -k\left({d}_{6}k-{c}_{7}+{k}^{2}{c}_{81}\right),$$

$${n}_{2}=k\left({d}_{7}k-{c}_{11}\right)\omega +k\left({d}_{4}+{c}_{9}k-{k}^{2}{d}_{5}\right),$$

$${n}_{3}=-{k}^{3}{c}_{82},$$

$${n}_{4}={{n}_{1}}^{2}-{{n}_{2}}^{2},$$

$${n}_{5}=2{n}_{1}{n}_{2},$$

$${n}_{6}=2{n}_{1}{n}_{3},$$

$${n}_{7}=2{n}_{2}{n}_{3},$$

$${n}_{8}={{n}_{3}}^{2},$$

$${B}_{1}={k}^{2}\left({c}_{12}k+{d}_{12}\right){\omega }^{2}-{k}^{3}\left({c}_{14}k+{d}_{10}\right)\omega +k\left({d}_{141}{k}^{3}+{c}_{16}-{k}^{2}{c}_{18}+\frac{{k}^{4}\sigma }{2}\right),$$

$${B}_{2}=k\left({c}_{13}+{d}_{11}k+{k}^{2}{c}_{15}\right)\omega +k\left(-{c}_{17}{k}^{3}+{d}_{13}-{k}^{2}{d}_{15}\right),$$

$${B}_{3}={d}_{142}{k}^{4},$$

$${p}_{1r}=-\left(\frac{{g}_{2}{\rho }_{2}}{{f}_{2}}-\frac{{g}_{1}{\rho }_{1}}{{f}_{1}}\right)\left({s}_{4}\left({{h}_{1}}^{2}-{{h}_{2}}^{2}\right)-2{s}_{5}{h}_{1}{h}_{2}\right)+{s}_{6}\left({s}_{4}{h}_{1}-{s}_{5}{h}_{2}\right)+{s}_{7}\left({h}_{2}{s}_{4}+{h}_{1}{s}_{5}\right)-\frac{1}{2}\left({s}_{4}{s}_{8}-{s}_{5}{s}_{9}\right),$$

$${p}_{2r}=2\left(\frac{{g}_{2}{\rho }_{2}}{{f}_{2}}-\frac{{g}_{1}{\rho }_{1}}{{f}_{1}}\right)\left({s}_{5}\left({h}_{1}{h}_{4}+{h}_{2}{h}_{3}\right)-{s}_{4}\left({h}_{1}{h}_{3}-{h}_{2}{h}_{4}\right)\right)+{s}_{7}\left({s}_{4}{h}_{4}+{s}_{5}{h}_{3}\right)-{s}_{6}\left({h}_{3}{s}_{4}-{h}_{4}{s}_{5}\right)-\frac{{s}_{4}{s}_{10}}{2}$$

$${p}_{3r}=-\left(\frac{{g}_{2}{\rho }_{2}}{{f}_{2}}-\frac{{g}_{1}{\rho }_{1}}{{f}_{1}}\right)\left({s}_{4}\left({{h}_{3}}^{2}-{{h}_{2}}^{2}\right)-2{s}_{5}{h}_{3}{h}_{2}\right),$$

$${p}_{1i}=-\left(\frac{{g}_{2}{\rho }_{2}}{{f}_{2}}-\frac{{g}_{1}{\rho }_{1}}{{f}_{1}}\right)\left({s}_{5}\left({{h}_{1}}^{2}-{{h}_{2}}^{2}\right)+2{s}_{4}{h}_{1}{h}_{2}\right)-{s}_{7}\left({s}_{4}{h}_{1}-{s}_{5}{h}_{2}\right)-{s}_{6}\left({h}_{2}{s}_{4}-{h}_{1}{s}_{5}\right)-\frac{1}{2}\left({s}_{4}{s}_{9}+{s}_{5}{s}_{8}\right),$$

$${p}_{2i}=-2\left(\frac{{g}_{2}{\rho }_{2}}{{f}_{2}}-\frac{{g}_{1}{\rho }_{1}}{{f}_{1}}\right)\left({s}_{4}\left({h}_{1}{h}_{4}+{h}_{2}{h}_{3}\right)+{s}_{5}\left({h}_{1}{h}_{3}-{h}_{2}{h}_{4}\right)\right)-{s}_{7}\left({s}_{4}{h}_{3}-{s}_{5}{h}_{4}\right)-{s}_{6}\left({h}_{4}{s}_{4}+{h}_{3}{s}_{5}\right)-\frac{{s}_{5}{s}_{10}}{2},$$

$${p}_{3i}=-\left(\frac{{g}_{2}{\rho }_{2}}{{f}_{2}}-\frac{{g}_{1}{\rho }_{1}}{{f}_{1}}\right)\left({s}_{5}\left({{h}_{3}}^{2}-{{h}_{2}}^{2}\right)+2{s}_{4}{h}_{3}{h}_{4}\right),$$

$${p}_{r}={p}_{1r}+{p}_{2r}{E}_{0}^{2}+{p}_{3r}{\left({E}_{0}^{2}\right)}^{2},$$

$${p}_{i}={p}_{1i}+{p}_{2i}{E}_{0}^{2}+{p}_{3i}{\left({E}_{0}^{2}\right)}^{2},$$

$${Q}_{1r}=3\left({B}_{1}{s}_{4}-{B}_{2}{s}_{5}\right)\left({{D}_{1}}^{2}+{{D}_{2}}^{2}\right)+2({D}_{1}\left({s}_{4}{n}_{4}-{s}_{5}{n}_{5}\right)+{D}_{2}\left({s}_{4}{n}_{5}-{s}_{5}{n}_{4}\right)),$$

$${Q}_{2r}=6{D}_{1}{D}_{3}\left({B}_{1}{s}_{4}-{B}_{2}{s}_{5}\right)+3{B}_{3}{s}_{4}\left({{D}_{1}}^{2}+{{D}_{2}}^{2}\right)+2({D}_{3}\left({s}_{4}{n}_{4}-{s}_{5}{n}_{5}\right)+{D}_{1}\left({s}_{4}{n}_{6}-{s}_{5}{n}_{7}\right)+{D}_{2}\left({s}_{4}{n}_{7}+{s}_{5}{n}_{6}\right)),$$

$${Q}_{3r}=6{D}_{1}{D}_{3}{B}_{3}{s}_{4}+3{{D}_{3}}^{2}\left({B}_{1}{s}_{4}-{B}_{2}{s}_{5}\right)+2(-{D}_{3}\left({s}_{5}{n}_{7}+{s}_{4}{n}_{6}\right)+{D}_{1}{s}_{4}{n}_{8}+{D}_{2}{s}_{5}{n}_{8}),$$

$${Q}_{4r}=3{{D}_{3}}^{2}{B}_{3}{s}_{4}+2{s}_{4}{n}_{8}{D}_{3},$$

$${Q}_{1i}=3\left({B}_{1}{s}_{5}+{B}_{2}{s}_{4}\right)\left({{D}_{1}}^{2}+{{D}_{2}}^{2}\right)+2({D}_{1}\left({s}_{4}{n}_{5}+{s}_{5}{n}_{4}\right)+{D}_{2}\left({s}_{4}{n}_{4}+{s}_{5}{n}_{5}\right)),$$

$${Q}_{2i}=6{D}_{1}{D}_{3}\left({B}_{1}{s}_{5}+{B}_{2}{s}_{4}\right)+3{B}_{3}{s}_{5}\left({{D}_{1}}^{2}+{{D}_{2}}^{2}\right)+2({D}_{3}\left({s}_{4}{n}_{5}+{s}_{5}{n}_{4}\right)+{D}_{1}\left({s}_{5}{n}_{6}+{s}_{4}{n}_{7}\right)+{D}_{2}\left({s}_{5}{n}_{7}-{s}_{4}{n}_{6}\right)),$$

$${Q}_{3i}=6{D}_{1}{D}_{3}{B}_{3}{s}_{5}+3{{D}_{3}}^{2}\left({B}_{2}{s}_{4}+{B}_{1}{s}_{5}\right)+2({D}_{3}\left({s}_{5}{n}_{6}+{s}_{4}{n}_{7}\right)+{D}_{1}{s}_{5}{n}_{8}-{D}_{2}{s}_{4}{n}_{8}),$$

$${Q}_{4i}=3{{D}_{3}}^{2}{B}_{3}{s}_{5}+2{s}_{5}{n}_{8}{D}_{3},$$

$${Q}_{r}={Q}_{1r}+{Q}_{2r}{E}_{0}^{2}+{Q}_{3r}{\left({E}_{0}^{2}\right)}^{2}+{Q}_{4r}{\left({E}_{0}^{2}\right)}^{3},$$

$${Q}_{i}={Q}_{1i}+{Q}_{2i}{E}_{0}^{2}+{Q}_{3i}{\left({E}_{0}^{2}\right)}^{2}+{Q}_{4i}{\left({E}_{0}^{2}\right)}^{3},$$

The constants of Eq. (62) may be listed as follows:

$${M}_{1}=3{{D}_{3}}^{2}{B}_{3}{s}_{5}+2{s}_{5}{n}_{8}{D}_{3},$$

$${M}_{2}=6{D}_{1}{D}_{3}{B}_{3}{s}_{5}+3{{D}_{3}}^{2}\left({B}_{2}{s}_{4}+{B}_{1}{s}_{5}\right)+2({D}_{3}\left({s}_{5}{n}_{6}+{s}_{4}{n}_{7}\right)+{D}_{1}{s}_{5}{n}_{8}-{D}_{2}{s}_{4}{n}_{8}),$$

$${M}_{3}=6{D}_{1}{D}_{3}\left({B}_{1}{s}_{5}+{B}_{2}{s}_{4}\right)+3{B}_{3}{s}_{5}\left({{D}_{1}}^{2}+{{D}_{2}}^{2}\right)+2({D}_{3}\left({s}_{4}{n}_{5}+{s}_{5}{n}_{4}\right)+{D}_{1}\left({s}_{5}{n}_{6}+{s}_{4}{n}_{7}\right)+{D}_{2}\left({s}_{5}{n}_{7}-{s}_{4}{n}_{6}\right)),$$

$${M}_{4}=3\left({B}_{1}{s}_{5}+{B}_{2}{s}_{4}\right)\left({{D}_{1}}^{2}+{{D}_{2}}^{2}\right)+2({D}_{1}\left({s}_{4}{n}_{5}+{s}_{5}{n}_{4}\right)+{D}_{2}\left({s}_{4}{n}_{4}+{s}_{5}{n}_{5}\right)),$$

The constants of Eq. (63) may be listed as follows:

$${N}_{1}={p}_{3r}{Q}_{4r}+{p}_{3i}{Q}_{4i},$$

$${N}_{2}={p}_{2r}{Q}_{4r}+{p}_{3r}{Q}_{3r}+{p}_{2i}{Q}_{4i}+{p}_{3i}{Q}_{3i},$$

$${N}_{3}={p}_{2r}{Q}_{3r}+{p}_{3r}{Q}_{2r}+{p}_{1r}{Q}_{4r}+{p}_{1i}{Q}_{4i}+{p}_{2i}{Q}_{3i}+{p}_{3i}{Q}_{2i},$$

$${N}_{4}={p}_{1r}{Q}_{3r}+{p}_{2r}{Q}_{2r}+{p}_{3r}{Q}_{1r}+{p}_{1i}{Q}_{3i}+{p}_{2i}{Q}_{2i}+{p}_{3i}{Q}_{1i},$$

$${N}_{5}={p}_{1r}{Q}_{2r}+{p}_{2r}{Q}_{1r}+{p}_{1i}{Q}_{2i}+{p}_{2i}{Q}_{1i},$$

$${N}_{6}={p}_{1r}{Q}_{1r}+{p}_{1i}{Q}_{1i},$$

The constants of Eq. (65) may be listed as follows:

$$l_{1} = \frac{{c_{2} }}{{c_{1} }},\,\,\,\,\,\,\,l_{3} = \frac{{c_{6} }}{{c_{1} }},$$

$${l}_{2}=\frac{\left({c}_{4}+\sigma {k}^{2}-k\left({d}_{31}+{d}_{32}{{E}_{0}}^{2}\right)\right)}{{c}_{1}},$$

$${l}_{4}=\frac{\left({c}_{7}-{d}_{6}k-{k}^{2}\left({c}_{81}+{c}_{82}{{E}_{0}}^{2}\right)\right)}{{c}_{1}},$$

$$l_{5} = - k\frac{{d_{9} }}{{c_{1} }},\,\,\,\,\,\,l_{6} = \frac{{\left( {c_{11} - kd_{7} } \right)}}{{c_{1} }},$$

$${l}_{7}=\frac{\left({c}_{16}+\frac{\sigma {k}^{4}}{2}+{k}^{3}\left({d}_{141}+{d}_{142}{{E}_{0}}^{2}\right)\right)}{{c}_{1}},$$

$${l}_{8}=-{k}^{2}\frac{{c}_{12}}{{c}_{1}},$$

$${l}_{9}=\frac{\left({c}_{13}-k{d}_{11}-{k}^{2}{c}_{15}\right)}{{c}_{1}},$$

$${l}_{10}=-k\frac{{d}_{12}}{{c}_{1}},$$

$${L}_{1}=\frac{\left({c}_{3}+{d}_{1}\right)}{{c}_{1}},$$

$${L}_{2}=\frac{\left(k{c}_{5}+{d}_{2}\right)}{{c}_{1}},$$

$${L}_{3}=\frac{\left(k{c}_{9}+{d}_{4}-{k}^{2}{d}_{5}\right)}{{c}_{1}},$$

$${L}_{4}=\frac{k\left(-k{d}_{8}+{c}_{10}\right)}{{c}_{1}},$$

$${L}_{5}=\frac{\left(-{k}^{3}{c}_{17}+{d}_{13}-{k}^{2}{d}_{15}\right)}{{c}_{1}},$$

$${L}_{6}=\frac{-{k}^{2}\left(k{c}_{14}+{d}_{10}\right)}{{c}_{1}},$$

$$\varpi =\frac{{l}_{2}{L}_{1}-{l}_{1}{L}_{2}}{{L}_{1}},$$

The constants of Eq. (66) may be listed as follows:

$${A}_{1}=\frac{{l}_{4}{L}_{1}^{2}-{l}_{6}{L}_{1}{L}_{2}+{l}_{3}{L}_{2}^{2}-{l}_{1}{L}_{1}{L}_{3}+2{l}_{2}{L}_{1}{L}_{4}-{l}_{1}{L}_{2}{L}_{4}}{{L}_{1}^{2}},$$

$${A}_{2}=\frac{{l}_{5}{L}_{1}^{2}+2{L}_{1}{L}_{4}}{{L}_{1}^{2}},$$

$${A}_{3}=\frac{1}{{L}_{1}^{2}}\left({l}_{7}{L}_{1}^{2}-{l}_{9}{L}_{1}{L}_{2}+{l}_{10}{L}_{2}^{2}-{l}_{6}{L}_{1}{L}_{3}+2{l}_{3}{L}_{2}{L}_{3}+2{l}_{4}{L}_{1}{L}_{4}-{l}_{6}{L}_{2}{L}_{4}-{l}_{1}{L}_{3}{L}_{4}+{l}_{2}{L}_{4}^{2}-{l}_{1}{L}_{1}{L}_{5}+ 2{l}_{2}{L}_{1}{L}_{6}-{l}_{1}{L}_{2}{L}_{6}\right),$$

$${A}_{4}=\frac{{l}_{8}{L}_{1}^{2}+2{l}_{5}{L}_{1}{L}_{4}+{L}_{4}^{2}+2{L}_{1}{L}_{6}}{{L}_{1}^{2}}.$$

## List of symbols

$R$: Undisturbed cylindrical radius
$\underline{\underline{A}}$: Rate of the strain tensor
$F(\eta )$: Net thermal flux via the interface
$F(0)$: Net thermal flux among the interface and fluid zones.
$$\underline{g}$$: Gravitational acceleration
$G$: A constant at the equilibrium state
$E_{0}$: Uniform axial electric field
$$I$$: Identity matrix
$k$: Wave number of the surface wave
$K_{1} ,\,K_{2}$: Heat conductivity at lower and upper fluids, respectively
$L$: Latent heat transmutation from one fluid to the other one
$$m$$: Porosity parameter
$\underline{n}$: Unit outward normal vector
$$t$$: Time
$$P$$: Hydrostatic pressure
$T_{0}$: Constant temperature at the interface
$T_{1} \,,\,\,T_{2}$: Uniform temperature of inner and outer fluids, respectively
$\underline{v} = (u,\,w)$: Filter velocity of the fluid
$V$: Uniform basic velocity
$$(r,0,z)$$: Cylindrical coordinates
$$\begin{gathered} A_{i} (t),\,\,B_{i} (t) \hfill \\ C_{i} (t),\,\,D_{i} (t) \hfill \\ \end{gathered}$$: Time-dependent constants
$S,\,\,\,U$: Stable and unstable regions, respectively
$\alpha_{1}$: Linear MHT factor
$\alpha_{2}$: Second-order mass and heat transfer coefficient
$\alpha_{3}$: Third-order mass and heat transfer coefficient
$$\Gamma_{j}$$: Integration constant
$\varphi$: Scalar potential of the velocity field
$\psi$: Scalar potential of the electric field
$\gamma (t)$: Arbitrary time-dependent function
$$\eta$$: Surface displacement
$$\lambda$$: Flow permeability
$$\varepsilon$$: Dielectric constant
$$\xi$$: Darcy coefficient
$$\rho$$: Density
$$\underline{\underline{\varsigma }}$$: Stress deviator
$\underline{\underline{\tau }}^{(elec)}$: Stress tensor electric field
$\underline{\underline{\tau }}^{(vis)}$: Cauchy stress tensor of Reiner–Rivlin fluid
$\beta$: Coefficient of cross viscosity
$\gamma$: Coefficients of viscosity
$\Lambda$: Small arbitrary parameter
$\omega$: Frequency related to the surface wave

## Subscript

$j = 1\,{\text{and}}\,2$: The inner and outer liquids
